# Application of Next-Generation Sequencing for Genetic Diagnosis in Neonatal Intensive Care Units: Results of a Multicenter Study in China

**DOI:** 10.3389/fgene.2020.565078

**Published:** 2020-11-06

**Authors:** Tianwen Zhu, Xiaohui Gong, Fei Bei, Li Ma, Yan Chen, Yonghong Zhang, Xia Wang, Jingjing Sun, Jian Wang, Gang Qiu, Jianhua Sun, Yu Sun, Yongjun Zhang

**Affiliations:** ^1^Department of Neonatology, Xinhua Hospital, Shanghai Jiao Tong University School of Medicine, Shanghai, China; ^2^Department of Neonatology, Shanghai Children’s Hospital, Shanghai Jiao Tong University School of Medicine, Shanghai, China; ^3^Department of Neonatology, Shanghai Children’s Medical Center, Shanghai Jiao Tong University School of Medicine, Shanghai, China; ^4^Department of Medical Genetics and Molecular Diagnostic Laboratory, Shanghai Children’s Medical Center, Shanghai Jiao Tong University School of Medicine, Shanghai, China; ^5^Department of Pediatric Endocrinology/Genetics, Xinhua Hospital, Shanghai Institute for Pediatric Research, Shanghai Jiao Tong University School of Medicine, Shanghai, China

**Keywords:** NICU, critically ill infants, phenotyping, next-generation sequencing, diagnostic procedure, genetic etiology

## Abstract

To identify next-generation-sequencing (NGS) clinical usability and to propose a standard diagnostic routine for critically ill infants, aged less than 100 days and suspected of having a genetically heterogeneous condition, a retrospective study was conducted between January 2016 and December 2018 at neonatal intensive care units (NICUs) of three tertiary hospitals in Shanghai, China. Whole-exome sequencing (WES) or panel sequencing was performed on 307 patients. Trio-WES, trio-panel, proband-WES, and proband-panel diagnostic yields were 39.71% (83/209), 68.75% (22/32), 59.09% (26/44), and 33.33% (4/12), respectively. Definitive molecular diagnoses of 142 infants (46.25%) uncovered 99 disorders; 21 disorders displayed on 44.37% of the diagnosed patients. Genetic etiologies were identified for 61.73% (50/81) of the deceased infants. One in three (29.58%) diagnosed infants exhibited one of the following four clinical traits which had a higher odds of diagnostic rate: integument abnormality (adjusted odds ratio [aOR], 19.7; 95% confidence interval [CI], 2.5–156.3), complex immune-related phenotypes (aOR, 9.2; 95% CI, 1.4–83.5), mixed nervous system phenotypes and congenital anomalies (aOR, 5.0; 95% CI, 1.3–19.1), or mixed metabolism and nervous system phenotypes (aOR, 4.5; 95% CI, 1.0–21.5). Our results demonstrated that NGS was an effective diagnostic tool. Infants exhibiting integument, complex immune-related conditions, metabolism, and nervous signs have higher chances of carrying variants in known disease-causing genes. The number of specific phenotypes could be used as an independent predictor of a positive molecular diagnosis, rather than an isolated abnormality. We developed a molecular diagnostic procedure for the use of NGS for diagnosis in Chinese NICU population based on individual characteristics.

## Introduction

With improvements in health care, genetic diseases have become the leading causes of infant mortality in neonatal intensive care units (NICUs) (Jacob et al., 2015). Understanding the genetic causes of the affected infants could help identify and improve potential therapeutic options (Fernandez-Marmiesse et al., 2018). Therefore, the need for genetic diagnoses of critically ill infants is paramount for the effective management of these patients (Tan et al., 2017; Gyngell et al., 2019). Furthermore, a timely diagnosis provides actionable information for a medical decision-making in the NICU (Willig et al., 2015; Wilkinson et al., 2016) and for their parents of reproductive ages (Vandersluis et al., 2020).

Genetics assays based on next-generation-sequencing (NGS) enable effective genome-wide detection of disease-causing variants. Although the value of NGS diagnostics in the intensive care setting is undisputed (Kapil et al., 2019), an optimal implementation strategy specifically for NICU populations has yet to be determined. Recently, several studies in Caucasian populations regarding the application of NGS in NICUs were reported (Daoud et al., 2016; Meng et al., 2017; French et al., 2019; Gubbels et al., 2019). However, these studies reached seemingly contradictory statements, some suggesting phenotype-driven selection (Gubbels et al., 2019) and the others in favor of a first-line strategy as a determiner in NGS assays (Stark et al., 2016; French et al., 2019). This controversy was primarily due to between-study heterogeneity depending on the country or region where the genetic studies are performed and overlapping clinical manifestations of genetic and non-genetic causes in neonatal/early infant period (Pogue et al., 2018).

China is a developing country with limited resources and low geneticist-to-population ratio (Hu et al., 2018). So a careful selection of patients who are eligible for NGS assays (Wilkinson et al., 2016) using sound procedures is imperative. In the current study, we systematically evaluated the utility of various NGS tests in 307 Chinese infants from NICUs at three institutions. The purpose of the study was to determine the molecular diagnostic yield, investigate the underlying genetic conditions, and develop an ideal molecular diagnostic work-flow for Chinese NICU population suspected with a genetic etiology.

## Materials and Methods

### Participating Institutions

This was a multicenter retrospective study of NGS findings for NICU patients at three tertiary hospitals in Shanghai, China. The institutions included Xinhua Hospital, Shanghai Jiao Tong University School of Medicine (XH); Children’s Hospital, Shanghai Jiao Tong University School of Medicine (CH); Shanghai Children’s Medical Center, Shanghai Jiao Tong University School of Medicine (SCMC). Three NICUs are level IV facilities. These units are regularly benchmarked against each other and have comparable outcomes with respect to disease spectrum, mortality and major morbidity. The analysis and publication of data related to the study were approved by the institutional review board at Xinhua Hospital, Shanghai Jiao Tong University School of Medicine (Approval number: XHEC-D-2019-101) with a waiver of consent and authorization.

### Patients

We collected patients who underwent NGS-based tests. The inclusion criteria are: (1) An age requirement of less than 100 days at the time of admission was used; (2) Clinical assessments of 180-day mortality (yes/no) were completed; (3) Parents provided written informed consent for the testing itself and agreed to pay for the NGS assay; (4) For patients from CH, the additional criteria were (i) referred as a suspected genetic condition by the treating neonatologists; (ii) recruited consecutively between Jun. 2017 (The time whole-exome sequencing (WES) was initiated in NICU) and Dec. 2018; (iii) WES was performed; For patients from XH and SCMC, the additional criteria were (i) admitted to the NICUs between Jan. 2016 and Dec. 2018; (ii) referred as a suspected genetic condition by a multidisciplinary group (the treating neonatologists and clinical geneticists); (iii) targeted exome sequencing (TES) panels were performed for patients based on the specific clinical phenotypes while WES was ordered for patients who presented with multiple congenital abnormalities or syndromic features or genetic conditions of no defined causes. The exclusion criteria are: (1) Patients with clear histories suggestive of a non-genetic cause; (2) Patients where a genetic diagnosis was already made; (3) Patients whose medical records could not provide complete information for this study; (4) The families who declined the 180-day of age telephone interviews; (5) Patients where a genetic diagnosis was made during the follow-up interval other than the initial TES panel or WES.

**FIGURE 1:**
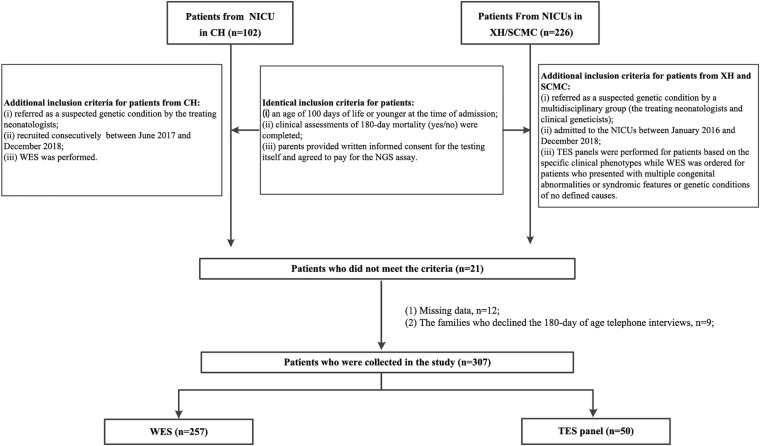
Patient flowchart. XH: Xinhua Hospital, Shanghai Jiao Tong University School of Medicine; CH, Children’s Hospital, Shanghai Jiao Tong University School of Medicine; SCMC, Shanghai Children’s Medical Center, Shanghai Jiao Tong University School of Medicine; NICU, Neonatal Intensive Care Unit; NGS, next-generation sequencing; TES, targeted exome sequencing; WES, whole-exome sequencing.

In total, 328 patients who meet the inclusion criteria were initially collected (102 from CH and 226 from XH and SCMC). Consequently, 307 eligible patients were included in the final analysis. A flowchart of the patients is shown in Figure 1. Data regarding gestational age, birth weight, family history of consanguinity, related clinical and molecular results were extracted from the medical records of the patients. The phenotypes of the affected infants were further translated into human phenotype ontology (HPO) terms (Kohler et al., 2017). Mortality outcome (yes or no) was obtained by telephone interview at six-month age.

### Genetic Sequencing and Data Analysis

DNA was extracted from the peripheral whole blood of 307 patients and their parents using a QIAamp Blood DNA Mini Kit (Qiagen GMBH, Hilden, Germany). For WES, the capture probes were those used in GenCap Custom Exome Enrichment Kits (MyGenostics, Beijing, China) and TruSight Rapid Capture Kits (Illumina, Inc., San Diego, CA, United States). Thirteen specific disease panels were used (MyGenostics) (see Supplementary Methods for details). Captured libraries were sequenced by an Illumina HiSeq 6000. The Burrows-Wheeler Aligner (BWA) (v.0.5.9-r16) was used to align the reads to the human reference genome (*GRCh37/hg19*). Copy number variations (CNVs) and small variants were identified using VarScan 2 and Genome Analysis Toolkit (GATK) (4.0.10.1).

Variants were interpreted and categorized according to the five-tier classification system recommended by the American College of Medical Genetics and Genomics (Richards et al., 2015; Brandt et al., 2020). Small variations were confirmed by Sanger sequencing. Potential CNVs identified by WES were further examined by karyotype testing or chromosomal microarray analysis (CMA). Multiplex ligation-dependent probe amplification (MLPA) analysis was performed to confirm deletions or duplications for Duchenne muscular dystrophy (DMD), neurofibromatosis, spinal muscular atrophy-1, and Prader-Willi syndrome (MyGenostics). All pathogenic, likely pathogenic variants have been deposited in Leiden Open Variation Database (LOVD) (see LOVD Individual IDs in Supplementary Table S1 for details).

Patients were considered to have a laboratory-confirmed genetic diagnosis if they had a pathogenic variant or likely pathogenic variant detected by a genetic test that explained the patient’s clinical presentation. For cases of autosomal recessive disorders, if one of the two variants were a variant of uncertain significance (VUS) presenting in combination with a pathogenic or likely pathogenic variant and if the phenotype appropriately matched, the VUS variants were also considered to be disease-causing.

### Statistical Analysis

The results are presented as means ± standard deviation (SD) for normally distributed continuous variables. Independent two-sample *t*-tests were used to compare normally distributed continuous variables between two groups. Continuous, but not normally distributed variables are presented as medians with their interquartile ranges (IQRs). The Mann–Whitney U test was used to compare two groups. Categorical variables are presented as frequencies. Comparison between categorical variables was performed using the chi-square test or Fisher’s exact test. All statistical tests were two-tailed and *P*-values < 0.05 were considered statistically significant. Data processing and statistical analyses were conducted using SPSS version 22.0 for Windows (IBM Corp, Armonk, NY, United States).

## Results

### Demographics and Clinical Indications for Testing

A total of 307 patients were collected, of which 124 were females (40.39%). 243 patients (79.15%) presented with disease onset during the neonatal period, while 64 (20.85%) presented after 28 days of age. Additional demographic characteristics of the patients are summarized in Table 1.

**TABLE 1:** Clinical data for 307 infants from three medical centers.

Characteristics	Findings
**Sex [*n* (%)]**	
Male	183 (59.61%)
Female	124 (40.39%)
**Gestational age (weeks) [*n* (%)]**	
>37 weeks	217 (70.68%)
37∼28 weeks	84 (27.36%)
<28 weeks	6 (1.96%)
**Birth weight [*n* (%)]**	
≥2500 g	230 (74.92%)
1500∼2500 g	56 (18.24%)
<1500 g	21 (6.84%)
Age at enrollment [*M*(P25∼P75)], days	8 (1-26.00)
≤28 days of age [*n* (%)]	243 (79.15%)
28∼100 days of age [*n* (%)]	64 (20.85%)
Length of stay [*M* (P25∼P75)], days	14 (8-30)
Prenatal findings [*n* (%)]	29 (9.45%)
Consanguity [*n* (%)]	2 (0.65%)
Family history [*n* (%)]	14 (4.56%)
Age of testing [*M* (P25∼P75)], days	20 (8-39)
Age of confirmed diagnosis [*M* (P25∼P75)], days	76 (55–100)
Turnaround time [*M* (P25∼P75)], days	52 (35–66)
**Mortality [*n* (%)]**	
NICU	27 (8.79%)
Post-NICU	54 (17.59%)
Total 180 days	81 (26.38%)
**NGS tests [*n* (%)]**	
Panels	50 (16.29%)
WES	257 (83.71%)
**Testing protocols [*n* (%)]**	
Proband-panels	12 (3.91%)
Trio-panels	32 (10.42%)
Proband-WES	44 (14.33%)
Trio-WES	209 (68.08%)
Dyads	10 (3.26%)

*NGS, next-generation sequencing; WES, whole-exome sequencing.*

**FIGURE 2:**
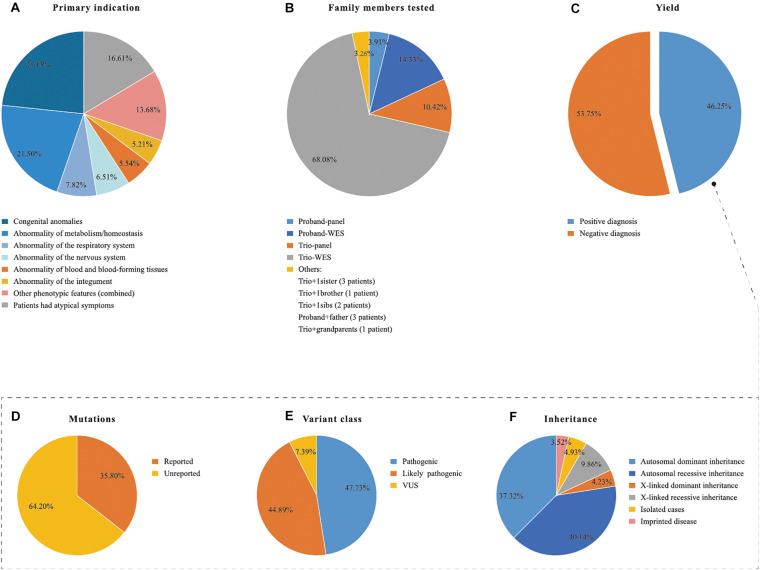
Descriptive statistics of the patient cohort and the positive NGS cases. NGS, next generation sequencing; TES, Targeted exome sequencing; WES, whole exome sequencing; VUS, variants of unknown clinical significance. **(A)** Primary indication, **(B)** family members tested, **(D–F)** in the dotted box explain the positive yield indicated in **(C)** in details.

Congenital anomalies (23.13%, 71/307) and suspected metabolic disorders (21.50%, 66/307) were the two most common indications of NGS requests, followed by abnormalities of the respiratory system (7.82%, 24/307), nervous system (6.51%, 20/307), blood and blood forming tissues (5.54%, 17/307), and integument (5.21%, 16/307). All other clinical features presented at low frequency, with each less than 5%. Fifty-one of the patients had atypical presentation (Figure 2A).

### Diagnostic Yield

An overall definitive molecular diagnosis of an established genetic disorder was provided for 142 of the 307 total cases (46.25%; Figure 2C). Panel analysis (*n* = 50, 50/307, 16.29%) and WES (*n* = 257, 257/307, 83.71%) were performed according to the clinical phenotypes of the patients (Table 1). Samples from both parents were available for 247 patients. Most patients underwent NGS under one of the following protocols: trio-WES (*n* = 209), trio-panels (*n* = 32), proband-WES (*n* = 44), or proband-panels (*n* = 12). 10 patients were submitted with dyads, depending on the family history and individual availability (Figure 2B). A molecular diagnosis was determined for 39.71% (83/209), 68.75% (22/32), 59.09% (26/44), 33.33% (4/12), and 70% (7/10) of the trio-WES, trio-panels, proband-WES, proband-panels, and dyads tests, respectively. In total, 177 clinically relevant variants were identified in 142 patients (Supplementary Table S1). Of the 177 variants, 84 were classified as pathogenic, 79 as likely pathogenic, and 13 as VUS (Figure 2D). Among the 177 variants, 113 were previously unreported (Figure 2E). The modes of inheritance were autosomal dominant (AD; 37.32%, 53/142), autosomal recessive (AR; 40.14%, 57/142), and X-linked (14.08%, 20/142) (Figure 2F). Five patients with Prader-Willi syndrome belonged to imprinted diseases (3.52%, 5/142), which were due to absence of paternally expressed imprinted genes at 15q11.2-q13 (Figure 2F).

**FIGURE 3:**
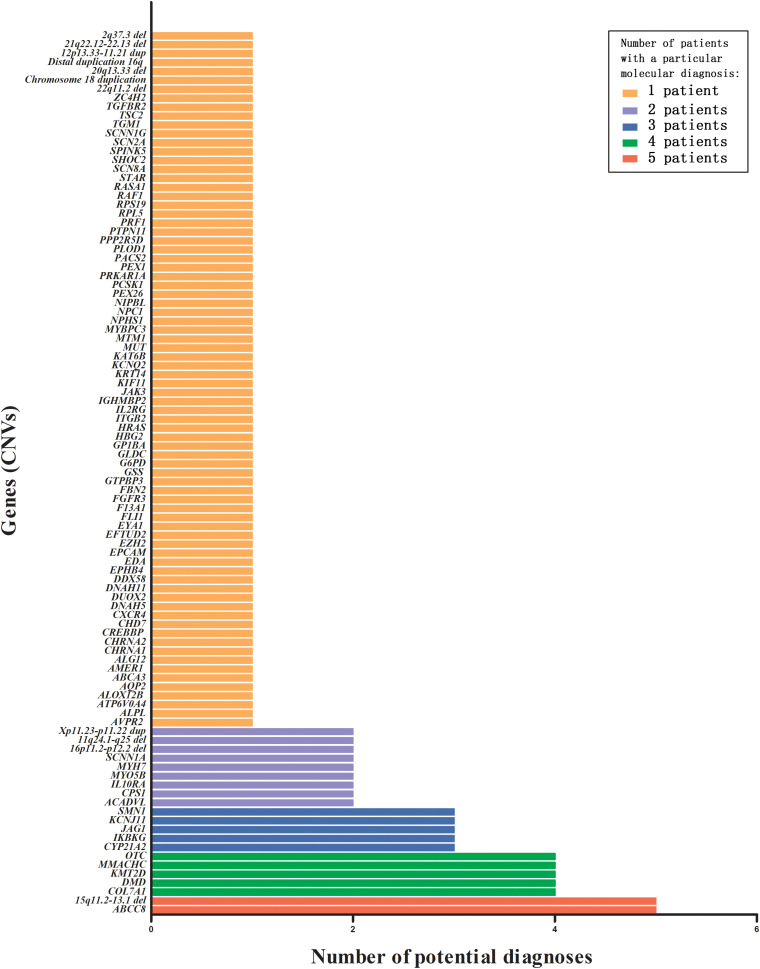
Number of potential diagnoses that were tested by various TES panels or WES, for all genes (including CNVs) with a pathogenic or likely pathogenic variant. TES, targeted exome sequencing; WES, whole-exome sequencing; CNV, copy number variations.

The spectrum of genetic diseases of our cohort is shown in Figure 3. A summary of the 99 disorders associated with 81 genes and 18 CNVs is provided in Supplementary Table S1. Twenty-one gene variants, including four CNVs, were identified in more than one proband. These included *ABCC8* (OMIM 162200) and 15q11q13 type 2 del in five infants each; *COL7A1* (OMIM 120120), *DMD* (OMIM 300377), *KMT2D* (OMIM 602113), *MMACHC* (OMIM 609831), and *OTC* (OMIM 300461) in four infants each; *CYP21A2* (OMIM 613815), *IKBKG* (OMIM 300248), *JAG1* (OMIM 601920), *KCNJ11* (OMIM 600937), and *SMN1* (OMIM 600354) in three infants each; *ACADVL* (OMIM 609575), *CPS1* (OMIM 608307), *IL10RA* (OMIM 146933), *MYH7* (OMIM 160760), *MYO5B* (OMIM 606540), *SCNN1A* (OMIM 600228), 16p12.2-p11.2 del, Xp11.23-p11.22 dup, and 11q24.1q25 del in two infants each. The remaining 78 gene variants, including 15 CNVs, were identified in one infant each.

### Spectrum Underlying 180-Day-Age Death

At 180-day-age follow-up, 81 of the 307 neonates died (26.38%) with 27 deaths (8.79%) occurring in the NICUs; 50 deceased patients received diagnoses based on NGS. The 180-day mortality rate was significantly higher in patients that had a molecular diagnosis (*n* = 50, 35.21%) in comparison with patients without a molecular diagnosis (*n* = 31, 18.79%; *P* = 0.001; Supplementary Table S2). As for the spectrum of genetic diseases for the 50 deceased infants that had a molecular diagnosis, 30 patients (60.00%) had lethal neonatal genetic diseases. We found that all of the patients diagnosed with *SMN1*-related spinal muscular atrophy-1 (OMIM 253300), very long-chain acyl-CoA dehydrogenase deficiency (OMIM 201475), microvillus inclusion disease (OMIM 251850), or *SCNN1A*-related Pseudohypoaldosteronism type I (OMIM 264350) died with each of these conditions being detected in at least two of the study cohort infants. On the other extreme of the disease spectrum for the deceased patients, we identified poor outcomes for certain nonlethal genetic diseases. *KMT2D*-related Kabuki syndrome 1 (OMIM: 147920) and *ABCC8*-related familial hyperinsulinemic hypoglycemia (OMIM 256450) were two of the most common disorders. Two infants with Kabuki syndrome died of serious complications following surgeries for their cardiovascular anomalies. The neonatal death of a preterm infant with familial hyperinsulinemic hypoglycemia was due to a serious sepsis/necrotizing enterocolitis. The medical records demonstrated that there were non-genetic contributory causes of death in these patients.

### Results According to Inclusion Scenarios and Clinical Phenotypic Categories

In this study data set, the inclusion criteria of CH differ from those of XH/SCMC. CH patients had earlier admission age [2(0.1–16) vs. 13(1–30.25), *P* < 0.001], younger GA (36.72 ± 3.62 vs. 37.82 ± 2.76, *P* = 0.019), low prenatal anomalies rate (1.05% vs. 13.21%, *P* = 0.002), no positive family history and earlier testing age [12(4–25) vs. 23(10–45.75), *P* < 0.001] as compared with patients in the other two hospitals. However, subgroup analysis revealed no significant difference between two inclusion scenarios for molecular diagnosis rate (44.21% vs. 47.17%, *P* = 0.631) and 180d mortality (29.47% vs. 25.00%, *P* = 0.411) (Supplementary Table S3).

Provided the main clinical indications for the NGS requests aforementioned, further robust investigation is warranted to assess whether specific clinical presentation were more likely to be associated with a molecular diagnosis (Table 2 and Supplementary Methods, Supplementary Tables S4 and S5). We found that the following four clinical traits had a higher odds of diagnostic rate: integument abnormality (*P* = 0.0048; aOR, 19.7; 95% CI, 2.5–156.3), abnormalities of the immune system (*P* = 0.0003; aOR, 9.2; 95% CI, 1.4–83.5), mixed phenotypes of nervous system and congenital anomalies (*P* = 0.0176; aOR, 5.0; 95% CI, 1.3–19.1), and mixed metabolism and nervous system phenotypes (*P* = 0.0495; aOR, 4.5; 95% CI, 1.0–21.5). One in three (29.58%) of the diagnosed infants exhibited one of above four clinical traits (Supplementary Table S5).

**TABLE 2:** Unadjusted and adjusted odds ratios of clinical phenotypes and mixed phenotypes for identification of individuals with a molecular diagnosis.

Phenotype composite index based on the number of systems involved	Crude OR (95% CI)	*P*-value	Adjusted OR (95% CI)	*P*-value
**Univariate analysis**				
One system affected				
Metabolism/homeostasis	3.3 (1.8, 5.8)	0.0001	2.8(1.5, 5.1)	**0.0008**
Nervous system	3.7 (1.7, 8.0)	0.0008	3.5(1.6, 7.7)	**0.0018**
Respiratory system	2.3 (1.3, 4.1)	0.0044	2.5(1.4, 4.5)	**0.0032**
Congenital anomalies	0.9 (0.5, 1.4)	0.5052	0.9(0.5, 1.4)	0.6028
Immune system	9.3(1.5, 85.2)	0.0001	9.2(1.4, 83.5)	**0.0003**
Integument	19.8(2.6, 151.5)	0.0041	19.7(2.5, 156.3)	**0.0048**
Blood and blood-forming tissues	3.3 (1.4, 8.3)	0.0089	3.6(1.4, 9.0)	**0.0070**
Cholestasis	0.6 (0.2, 1.7)	0.3266	0.7(0.2, 2.0)	0.4624
Cardiovascular system	1.7 (0.6, 4.7)	0.2723	1.6(0.6, 4.4)	0.3526
**Multivariate analysis**				
Two systems affected				
Metabolism/homeostasis and nervous system	6.3(1.4, 29.2)	0.0188	4.5(1.0, 21.5)	**0.0495**
Nervous system and congenital anomalies	4.2(1.1, 15.5)	0.0326	5.0(1.3, 19.1)	**0.0176**

*CI = confidence interval; OR, odds ratio; significance considered when *P* < 0.05; Adjusted OR for gestation age (GA) and birth weight (BW) of the patient (see additional file: Supplementary Methods, Supplementary Tables S4 and S5). Significant *p* values are highlighted in bold.*

### Derivation of the Predictors for a Molecular Diagnosis

Finally, a multivariate logistic regression was utilized to find out whether the specific phenotypes or the complexity of a phenotype of a patient (reflected by the number of HPO terms) could predict a molecular diagnosis. We identified that hypotonia, metabolic abnormality, skin anomaly, immune-pathological phenotype and a higher number of HPO terms could be used as independent predictors of molecular diagnosis (Supplementary Methods and Supplementary Table S6). Then, we analyzed ROC curve to verify the efficiency of above five variables and to determine their cutoff values that differentiated patients with and without a molecular diagnosis (Supplementary Methods for details). The AUC value for the “the number of HPO terms” was 0.777 (95% confidence interval (95%CI) = 0.726–0.829), which was better than those of four individual specific phenotypes (Supplementary Table S7). The optimal cutoff level of the HPO term number was set at more than 1.5. Since the HPO term number ranged from 0 to 6, with a whole number indicating how many phenotypes involved, we used “2” as the cutoff, which suggesting that two or more phenotypes are associated with a higher diagnostic yield (Supplementary Table S8 and Supplementary Figure S1).

## Discussion

Our study pooled data from multiple facilities to identify the followings for 307 patients in various NICUs: (1) underlying genetic conditions, (2) associated clinical indications for NGS, and (3) the 180-day-old outcomes for these infant patients. With that, our work demonstrated the feasibility of NGS-based tests in a difficult-to-diagnose patient population for whom the assay is most likely to be performed.

The high diagnostic rate and 180-day mortality rate derived from this work proved that NGS was an effective tool for critically ill infants suspected of a genetic disorder (French et al., 2019; Australian Genomics Health Alliance Acute Care Flagship et al., 2020; Śmigiel et al., 2020). Firstly, our diagnostic yield was similar to those reported in Western countries; with diagnostic rates of 36–57% (Willig et al., 2015; Meng et al., 2017; Powis et al., 2018; Brunelli et al., 2019; French et al., 2019) even though there were differences among the disease spectrum. Furthermore, the high mortality in our patients who died prior to six months of age (61.73%, 50/81) indicated that genetic diseases were the common causes in early life death. This is consistent with previous studies (Jacob et al., 2015; Wojcik et al., 2018; Marshall et al., 2019). These findings explicitly established that NGS should be used as an unbiased diagnostic option for infants in NICUs (Matthijs et al., 2016; Sawyer et al., 2016) in China.

Although some genetic diseases exhibit themselves within the first 28 days of life or shortly thereafter, their clinical symptoms are undifferentiated especially in those critically ill patients (French et al., 2019). In this study we found that some phenotypic groups had increased likelihoods of a positive diagnosis. Case in point, based on top-level HPO category analysis, patients with abnormalities in “metabolism/homeostasis,” “the nervous system,” “the respiratory system,” “the integument,” “immune system,” or “blood and blood-forming tissues” were significantly more likely to result in a genetic diagnosis. This is somewhat in agreement with the findings of Meng et al. (2017) who showed a higher diagnostic rate for abnormalities of the “musculature,” “blood, and blood-forming tissues,” and “metabolism/homeostasis” in their study. Furthermore, analysis of HPO category composition allowed us to determined that cases were referred due to mixed phenotypes involving congenital anomalies and nervous system, mixed phenotypes of metabolism/homeostasis and nervous system. We thus infer that these conditions were likely to result in a genetic diagnosis.

Given the retrospective nature of the study, various panels and WES were performed mainly as trios or proband. We found no significant difference in the diagnostic rate between trio-panel and proband-panel. However, a significant difference existed between trio-WES and proband-WES, with proband-WES associating with a higher molecular diagnostic yield. We can offer no obvious explanation for these data. The data limitation is a possibility, such as the sample size and personal preferences of neonatologists for NGS requests (Sun et al., 2015). The heterogeneity of phenotype spectrum is not an unlikely alternative. To elucidate the underlying possibility with phenotypes, we analyzed the diagnostic rates for these aforementioned trio-NGS tests and proband-NGS tests in some phenotypic subgroups. One of the most common phenotype of our cohort was metabolic abnormalities (21.50%, 66/307). For this category, the overall diagnostic rate is 68.18% (45/66); trio-panel was requested for 17 cases, proband-panel 4 cases, trio-WES 36 cases, and proband-WES 9 cases correspondingly. There was no significant improvement in the diagnostic yield of trio-panel, with 15 of 17 cases receiving a molecular diagnosis relative to the proband-panel cases (50.00%, 2/4). And there was not a significant improvement in the diagnostic yield of trio-WES relative to the proband-WES for this phenotypic subgroup. The results above suggest that a larger proportion of all possible genes for the family of metabolic disorders have been described. In contrast, referred patients with congenital anomalies, another common phenotype subgroup, had much lower diagnostic rates (32.39%, 23/71). We did not find significant difference in the diagnostic yield between trio-panels and proband-panels, or between trio-WES and proband-WES for this category. The results suggest a larger fraction of unknown genes underlying this subgroup and the strategy of simultaneous sequencing both parents and their affected infants appears not to offer much help. Thus it should be taken into consideration which sequencing method should be used in specific settings.

Finally and importantly, the key question is not whether to use genomic testing, but how it is used. Based on our study, there were some differences in the inclusion criteria between the patients from CH and those from XH/SMCM. The inclusion criteria required all eligible cases to be critically ill infants with a suspected, undiagnosed genetic condition. Although two groups differed in the NGS option and in whether a pre-test genetic consultation was performed, no significant difference in the diagnostic rate could be established. This finding indicated that a careful selection of patients by the clinical experiences of the treating physicians in NICU is an important strategy for implementation of genomic testing. Additionally, we identified that two or more specific phenotypes are associated with a higher diagnostic yield. This finding provided evidence to elucidate the relationship between complexity of the phenotypes of a patient and the expected diagnostic yield. In another study, Trujillano et al. (2017) showed that the diagnostic yield was remarkably higher (33%) in cases with 6–15 HPO terms and as much as 39% in cases with over 15 HPO terms. The difference might be associated with the significant between-cohort inclusion. Our patients were a selected group of critically ill infants, aged less than 100 days of age, whose symptoms have not fully presented to make a clear clinical diagnosis. Their cohort was composed of patient of all ages, with adults whose clinical manifestations were fully-expressed as the majority of the cohort. As a consequence, diagnostic approaches which are appropriate for adults and children cannot accommodate the special cases involving newborns. In order to better-identify genetic diseases in this population, we suggests a more permissive diagnostic algorithm.

**FIGURE 4:**
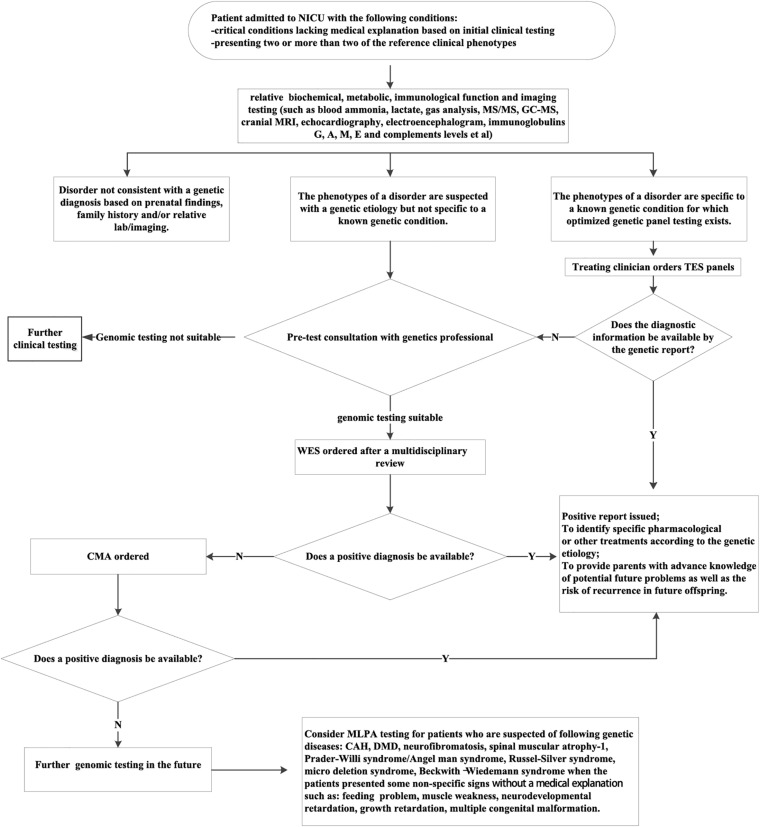
The proposed molecular diagnostic procedure to identify the genetic etiology of critically ill patients from NICUs. CAH, congenital adrenal cortical hyperplasia; CMA, chromosomal microarray analysis; DMD, Duchenne muscular dystrophy; MLPA, multiplex ligation-dependent probe amplification; TES, targeted exome sequencing; WES, whole-exome sequencing. The reference clinical phenotypes includes complex metabolic phenotypes (e.g., persistent hypoglycemia, hyperkalemia, hyponatremia, metabolic acidosis, lactic acidosis, and hyperammonemia); unexplained neurological signs (e.g., seizures and hypotonia); unexplained respiratory failure; unexplained abnormalities of the cardiovascular system (e.g., cardiomyopathy and arrhythmia); skin lesions of unknown origin(e.g., ichthyosis and blister); unexplained thrombocytopenia or anemia; congenital malformations that are not consistent with any known syndrome; unexplained abnormalities of the immune system(e.g., recurrent infections, protracted diarrhea, leukocytosis, leukopenia, and abnormal immunoglobulin level); unexplained cholestasis.

This new molecular diagnostic procedure (Figure 4) could be applied in NICUs. Patients suspected of a genetic etiology who recently exhibited two or more of the reference phenotypes were considered as potential candidates for NGS assays. Their diagnostic information could not be attained through conventional biochemical, metabolic, and imaging testing. When the patient’s phenotype is specific to a known genetic condition, for which an optimized genetic panel testing exists, the targeted gene panel should be given. When the phenotype of the patient is not specific to a known genetic condition, WES was recommended. Given the large proportion of *de novo* diagnostic variants and novel ones derived from our study (Supplementary Table S1), it is plausible to suggest trio-tests that are more likely to receive a diagnosis. Nevertheless, NGS/CMA would not solve every case. The negative cases need further investigation; and the MLAP analysis is an option in situations in which the corresponding pathogeneses of the genetic diseases are related to the presence of deletions or duplications or abnormal DNA methylation of specific genes (Stuppia et al., 2012).

Our study has several limitations. A potential limitation was that our work did not include management details and the economic issue. Therefore, it remains unknown to what extent NGS may affect the personalized treatments and shorten the time of hospitalization for patients if NGS-based tests would have been used as a first-tier test after their admissions (Tan et al., 2017; Farnaes et al., 2018; Stark et al., 2018). Second, there was a methodological heterogeneity in inclusion criteria for patients among three participating hospitals, which might potentially impact our results. Third, the diagnostic use of NGS failed to detect all causal variant types, resulting in specific variants not being identified. Fourth, while our study was a multicenter retrospective study, three institutions are affiliated to Shanghai Jiao Tong University School of Medicine and they represent the highest level of NICU expertise in developed regions across China. Thus, the ability to generalize our results to the genetic diagnosis yield may be somewhat tenuous. We hope to confirm our findings with a prospective research in the future.

## Conclusion

To our knowledge, this is the first large-scale multicenter studies concerning NGS application in NICU patients in China. We propose a molecular diagnostic procedure based on individual characteristics for Chinese NICU population with complex traits.

## Data Availability Statement

The databases used and/or analyzed during the current study are available from the corresponding author on reasonable request. All data relevant to the study are included in the article/Supplementary Material. All pathogenic, likely pathogenic variants have been deposited in Leiden Open Variation Database (LOVD) (see LOVD Individual IDs in Supplementary Table S1 for details).

## Ethics Statement

The studies involving human participants were reviewed and approved by Ethics Committee of Xin Hua Hospital Affiliated to Shanghai Jiao Tong University School of Medicine (Approval number: XHEC-D-2019-101). Written informed consent to participate in this study was provided by the participants’ legal guardian/next of kin.

## Author Contributions

YJZ and YS conceived and designed the study. TZ, XG, and FB prepared an analytical plan, analyzed the data, and drafted the initial manuscript. YS and YJZ collaborated in the revision and interpretation of the data and results, and revised the manuscript. LM, YC, YHZ, XW, JJS, JW, GQ, and JHS involved in the data collection, manuscript review, and revision. All authors commented on the manuscript and approved the final manuscript as submitted.

## Conflict of Interest

The authors declare that the research was conducted in the absence of any commercial or financial relationships that could be construed as a potential conflict of interest.
