# Supplementary material for: Application of Next-Generation Sequencing for Genetic Diagnosis in Neonatal Intensive Care Units: Results of a Multicenter Study in China
Source: Front Genet. 2020 Nov 6;11:565078. doi: 10.3389/fgene.2020.565078 (PMC7677510; doi:10.3389/fgene.2020.565078)
Supplement: Supplementary file 1 [file Data_Sheet_1.PDF]

Supplementary Figure

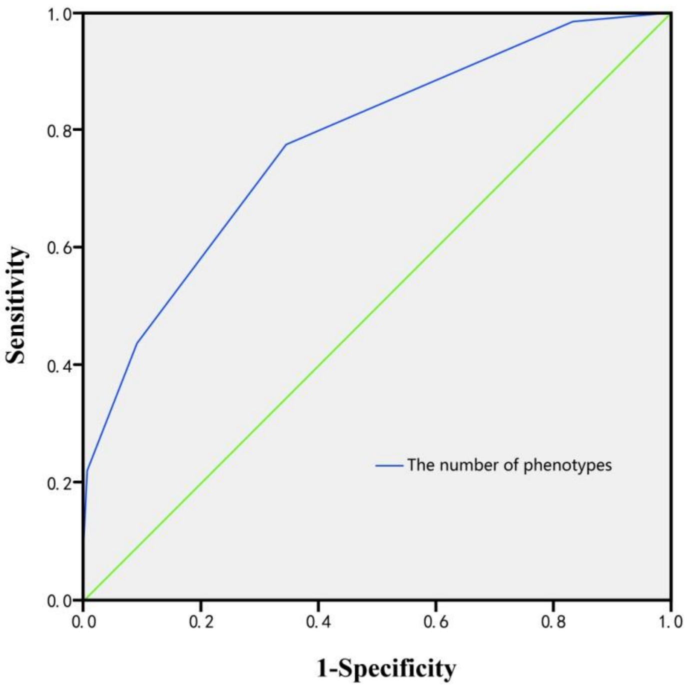

Figure S1. Receiver operating characteristic (ROC) curve for "the number of phenotypes".
