# Supplementary material for: Application of Next-Generation Sequencing for Genetic Diagnosis in Neonatal Intensive Care Units: Results of a Multicenter Study in China
Source: Front Genet. 2020 Nov 6;11:565078. doi: 10.3389/fgene.2020.565078 (PMC7677510; doi:10.3389/fgene.2020.565078)
Supplement: Supplementary file 2 [file Data_Sheet_2.docx]

**SUPPLEMENTARY MATERIAL**

Supplementary file includes:

1. Supplementary Methods: a section about the list of thirteen (TES panels (designed by MyGenostics, Beijing, China) for 50 patients in our cohort; prediction of phenotypes or mixed phenotypes for identification of individuals with a molecular diagnosis.

(2) Supplementary Tables: Tables S1, S2, S3, S4, S5, S6, S7, S8.

**Supplementary Methods**

**List of the 13 (TES panels (designed by MyGenostics, Beijing, China) for 50 patients in our cohort.**

| Name for TES Panels | Number of targeted genes involved | Tested Number of patents |
| --- | --- | --- |
| Neonatal metabolism panel | 175 | 14 |
| Mitochondrial gene panel | 37 | 2 |
| Endocrine panel | 108 | 6 |
| DMD panel | 1 | 3 |
| SMA panel | 2 | 1 |
| Epilepsy panel | 534 | 3 |
| Hematological system diseases panel | 277 | 4 |
| Cardiovascular system panel | 902 | 3 |
| Digestive system panel | 54 | 7 |
| Dermatological disease panel | 442 | 4 |
| Nephrosis panel | 44 | 1 |
| Immune panel | 232 | 1 |
| Ophthalmic disease panel | 193 | 1 |

Abbreviation: TES, Targeted Exome Sequencing; DMD, Duchenne muscular dystrophies; SMA, Spinal Muscular trophy.

**List of all covered genes in the 13 TES panels**

| Name for TES Panels | Genes involved in the panel | | | | | | | | | |
| --- | --- | --- | --- | --- | --- | --- | --- | --- | --- | --- |
| Neonatal metabolism panel |  | | | | | | | | | |
|  | *PAH* | *L2HGDH* | *DHTKD1* | *CTH* | *ARX* | *ATP7B* | *GCSH* | *PTS* | *D2HGDH* | *GALK1* |
|  | *MTHFR* | *SLC6A8* | *MMAA* | *G6PD* | *CPS1* | *GCH1* | *IDH2* | *INPP5E* | *MTRR* | *GAMT* |
|  | *MMAB* | *ATP7A* | *OTC* | *QDPR* | *ETFA* | *LAMP2* | *MTR* | *GATM* | *ABCD4* | *PTPN11* |
|  | *ASS1* | *PCBD1* | *ETFB* | *MAOA* | *GNMT* | *ERCC8* | *GPHN* | *MVK* | *ASL* | *SPR* |
|  | *ETFDH* | *PNPLA2* | *AHCY* | *ERCC6* | *MCEE* | *ACSF3* | *ARG1* | *FAH* | *BCAT1* | *SLC2A1* |
|  | *SLC25A13* | *OGDH* | *OAT* | *TAT* | *BCAT2* | *HSD17B10* | *HCFC1* | *FH* | *NAGS* | *HPD* |
|  | *SLC22A5* | *SLC2A2* | *GLUD1* | *FOLR1* | *LMBRD1* | *AASS* | *SLC7A7* | *HGD* | *CPT1A* | *SLC3A1* |
|  | *GLUL* | *FOLR2* | *GCDH* | *ABHD5* | *MAT1A* | *HAL* | *CPT2* | *SLC7A9* | *BCKDHA* | *DHFR* |
|  | *HMGCL* | *ACAT1* | *CBS* | *UROC1* | *SLC25A20* | *BCKDHB* | *DDC* | *AUH* | *ADK* | *SUOX* |
|  | *FTCD* | *MLYCD* | *TYMP* | *DBT* | *PHGDH* | *TAZ* | *ALDH6A1* | *MOCS1* | *GLDC* | *ACADSB* |
|  | *TK2* | *DLD* | *PSAT1* | *OPA3* | *ASPA* | *MOCS2* | *AMT* | *ACADS* | *DGUOK* | *SARDH* |
|  | *ABAT* | *SERAC1* | *DBH* | *NR0B1* | *ACADM* | *POLG* | *PRODH* | *ALDH5A1* | *FBXL4* | *MCCC2* |
|  | *SOX9* | *ACADVL* | *SUCLA2* | *ALDH4A1* | *SRY* | *ALPL* | *KMT2D* | *PCCA* | *CYP21A2* | *HADHA* |
|  | *MPV17* | *SLC6A20* | *AR* | *PNPO* | *KDM6A* | *PCCB* | *CYP11B1* | *HADHB* | *C10orf2* | *SLC6A19* |
|  | *HSD17B3* | *ETHE1* | *SGSH* | *HLCS* | *HSD3B2* | *HADH* | *RRM2B* | *SLC36A2* | *SRD5A2* | *FOXG1* |
|  | *NAGLU* | *BTD* | *CYP17A1* | *ACAD8* | *SUCLG1* | *IVD* | *NR5A1* | *MECP2* | *HGSNAT* | *PC* |
|  | *StAR* | *TH* | *SLC25A4* | *MCCC1* | *WT1* | *CDKL5* | *GNS* | *MUT* | *ALDH7A1* | *GLYCTK* |
|  | *MMADHC* | *SLC46A1* | *SLC19A1* | *SLC25A15* | *MMADHC* |  |  |  |  |  |
| Endocrine panel |  |  |  |  |  |  |  |  |  |  |
|  | *ABCC8* | *EPO* | *GLUD1* | *IER3IP1* | *PAX6* | *SLC16A1* | *ZFP57* | *AKT2* | *FOXP3* | *HADH* |

**List of all covered genes in the thirteen TES panels**

| Name for TES Panels | Genes involved in the panel | | | | | | | | | |
| --- | --- | --- | --- | --- | --- | --- | --- | --- | --- | --- |
|  | *CYP2R1* | *GATA3* | *MINPP1* | *PCM1* | *SLC16A2* | *TRH* | *APOA1* | *DICER1* | *GCM2* | *MYH8* |
|  | *IL1RN* | *MNX1* | *PDX1* | *SLC19A2* | *ALG3* | *GATA4* | *HFE* | *INS* | *NEUROD1* | *PLAGL1* |
|  | *SLC2A2* | *BLK* | *GATA6* | *HNF1A* | *INSR* | *NEUROG3* | *PON1* | *SOD2* | *CEL* | *GCK* |
|  | *HNF1B* | *KCNJ11* | *NKX2-2* | *PTF1A* | *UCP2* | *EIF2AK3* | *GLIS3* | *HNF4A* | *KLF11* | *PAX4* |
|  | *RFX6* | *VEGFA* | *MAPK8IP1* | *ABCA1* | *CLEC7A* | *FOXE1* | *IYD* | *NTRK1* | *SDHD* | *THRB* |
|  | *VDR* | *AIRE* | *CYP27B1* | *FOXP3* | *MEN1* | *PAX8* | *SECISBP2* | *TPO* | *ZFAT* | *ALB* |
|  | *PHEX* | *SLC26A4* | *TRIM24* | *BCAM* | *DMP1* | *GLIS3* | *NCOA4* | *PRKAR1A* | *TRIM33* | *CACNA1S* |
|  | *GNAS* | *NDUFA13* | *PTEN* | *SLC5A5* | *TSHB* | *CASR* | *DUOXA2* | *NKX2-1* | *PTH* | *TBCE* |
|  | *TSHR* | *CDC73* | *ENPP1* | *HRAS* | *NKX2-5* | *RET* | *TG* | *TTR* | *CLCN5* | *FGF23* |
|  | *IGSF1* | *NRAS* | *SDHB* | *THRA* | *UBR1* | *SLC34A3* | *GOLGA5* | *DUOX2* |  |  |
| DMD panel |  |  |  |  |  |  |  |  |  |  |
|  | *DMD* |  |  |  |  |  |  |  |  |  |
| SMA panel |  |  |  |  |  |  |  |  |  |  |
|  | *SMN1* | *SMN2* |  |  |  |  |  |  |  |  |
| Digestive system panel |  |  |  |  |  |  |  |  |  |  |
|  | *NEUR0G3* | *SPINT2* | *SLC26A3* | *EPCAM* | *MY05B* | *CTLA4* | *MY09B* | *BSND* | *APC* | *HLA-DQB1* |
|  | *ABCG8* | *ABCB4* | *PRKCSH* | *SEC63* | *CIRH1A* | *KRT8* | *KRT18* | *IRGM* | *ADAM17* | *ATG16L1* |
|  | *ABCB1* | *IRF5* | *IL23R* | *IL10RB* | *IL10RA* | *NOD2* | *IL6* | *PRSS1* | *SPINK 1* | *CFTR* |
|  | *RET* | *ECE1* | *EDNRB* | *GDNF* | *EDN3* | *NRTN* | *SMAD7* | *IFNGR1* | *GIF* | *UGT1A1* |
|  | *TRMU* | *FLNA* | *ABCC2* | *NCR3* | *TYMP* | *TRIM37* | *SALL1* | *SLC5A1* | *ACVRL1* | *SLC46A1* |
|  | *ALDOB* | *MKKS* | *SAR1B* | *SCN9A* | *ALDOB* |  |  |  |  |  |

**List of all covered genes in the thirteen TES panels**

| Name for TES Panels | Genes involved in the panel | | | | | | | | | |
| --- | --- | --- | --- | --- | --- | --- | --- | --- | --- | --- |
| Epilepsy panel |  |  |  |  |  |  |  |  |  |  |
|  | *ABAT* | *BCKDK* | *CTSA* | *FASTKD2* | *HNF1B* | *MDGA2* | *OPHN1* | *RAB3GAP1* | *ABCC2* | *BCS1L* |
|  | *CTSD* | *FCGR2B* | *HNRNPH1* | *ME2* | *PAFAH1B1* | *RAF1* | *ABCC8* | *BOLA3* | *CTSF* | *FGD1* |
|  | *HNRNPU* | *MECP2* | *PAH* | *RANBP2* | *ACADSB* | *BRAF* | *CUL4B* | *FGF8* | *HP* | *MED12* |
|  | *PAK3* | *RARS2* | *ACOX1* | *BSN* | *CYB5R3* | *FGFR3* | *HPD* | *MEF2C* | *PANK2* | *RBFOX1* |
|  | *ACTB* | *BTD* | *CYP2R1* | *FH* | *HRAS* | *MFSD8* | *PAX6* | *RBFOX2* | *ACY1* | *TWNK* |
|  | *CYP2U1* | *FKRP* | *HSD17B10* | *MGAT2* | *PC* | *RBFOX3* | *COQ8A* | *C12orf65* | *D2HGDH* | *FKTN* |
|  | *HSD17B4* | *MLC1* | *PCDH19* | *RELN* | *ADK* | *C4A* | *DAO* | *FLNA* | *HTR2A* | *MMACHC* |
|  | *PCNT* | *RFT1* | *ADSL* | *CACNA1A* | *DAOA* | *FOLR1* | *HTT* | *MOCS1* | *PDHA1* | *RNASEH2A* |
|  | *AFG3L2* | *CACNA1H* | *DBH* | *FOXG1* | *HYAL1* | *MOCS2* | *PDHX* | *RNASEH2B* | *AGA* | *CACNB4* |
|  | *DBT* | *FOXP2* | *IDH2* | *MOCS3* | *PDSS1* | *RNASEH2C* | *AHI1* | *CACNG2* | *DCX* | *FOXRED1* |
|  | *IDS* | *MOGS* | *PDSS2* | *ROGDI* | *AKT1* | *CASK* | *DDC* | *FUCA1* | *IDUA* | *MPC1* |
|  | *PEX1* | *RPGRIP1L* | *ALDH4A1* | *CASR* | *DDOST* | *GABBR2* | *IER3IP1* | *MPDU1* | *PEX10* | *RPIA* |
|  | *ALDH5A1* | *CC2D2A* | *DEPDC5* | *GABRA1* | *IFNG* | *MPI* | *PEX12* | *RTN4R* | *ALDH7A1* | *CDH13* |
|  | *DGKD* | *GABRA6* | *IL6* | *MR1* | *PEX13* | *RYR1* | *ALG1* | *CDH9* | *DHCR7* | *GABRB2* |
|  | *INPP5E* | *MTHFR* | *PEX14* | *RYR3* | *ALG11* | *CDKL5* | *DHFR* | *GABRB3* | *INS* | *MTOR* |
|  | *PEX16* | *SAMHD1* | *ALG12* | *CEP152* | *DIAPH3* | *GABRD* | *IQSEC2* | *MTR* | *PEX19* | *SCARB2* |
|  | *ALG13* | *CEP290* | *DISC1* | *GABRG2* | *KCNA1* | *MTRR* | *PEX2* | *SCN1A* | *ALG2* | *CHD2* |
|  | *DLD* | *GALC* | *KCNB1* | *NAGLU* | *PEX26* | *SCN1B* | *ALG3* | *CHI3L1* | *DMPK* | *GALNS* |
|  | *KCNH5* | *NDE1* | *PEX3* | *SCN2A* | *ALG6* | *CHRNA2* | *DNAJC5* | *GAMT* | *KCNJ1* | *NDN* |

**List of all covered genes in the thirteen TES panels**

| Name for TES Panels | Genes involved in the panel | | | | | | | | | |
| --- | --- | --- | --- | --- | --- | --- | --- | --- | --- | --- |
|  | *EHMT1* | *GPC3* | *LBR* | *NDUFV2* | *POMGNT1* | *SLC20A2* | *ASPA* | *COG5* | *EIF2B1* | *GPHN* |
|  | *PHF6* | *SERPINI1* | *APOL2* | *CLCN2* | *DOLK* | *GFAP* | *KCNQ3* | *NDUFAF3* | *PHGDH* | *SETBP1* |
|  | *APOL4* | *CLCN4* | *DPAGT1* | *GLB1* | *KCNT1* | *NDUFAF4* | *PIGA* | *SGCE* | *APP* | *CLCNKA* |
|  | *DPM1* | *GLDC* | *KCTD7* | *NDUFAF5* | *PIGL* | *SGSH* | *APTX* | *CLCNKB* | *DPM3* | *GLRA1* |
|  | *KDM5C* | *NDUFB3* | *PIGV* | *SHANK3* | *ARG1* | *CLN3* | *DPYD* | *GLRB* | *KIF11* | *NDUFS1* |
|  | *PLA2G6* | *SHH* | *ARHGAP3* | *CLN5* | *DRD2* | *GLUD1* | *KIF1A* | *NDUFS2* | *PLCB1* | *SHOC2* |
|  | *ARHGEF9* | *CLN6* | *DRD3* | *GLUL* | *KMT2D* | *NDUFS3* | *PLP1* | *SIX3* | *ARL13B* | *CLN8* |
|  | *DTNBP1* | *GNE* | *KRAS* | *NDUFS4* | *PMM2* | *SLC13A5* | *ARSA* | *CNTN5* | *EBP* | *GNPTAB* |
|  | *PEX5* | *SCN4A* | *ALG8* | *CHRNA3* | *DNAJC6* | *GATM* | *KCNJ10* | *NDUFA1* | *PEX6* | *SCN8A* |
|  | *ALG9* | *CHRNA4* | *DNASE1* | *GBA* | *KCNJ11* | *NDUFA11* | *PEX7* | *SCN9A* | *AMACR* | *CHRNA5* |
|  | *DNM1* | *GCDH* | *KCNMA1* | *NDUFA2* | *PGK1* | *SCO2* | *AMER1* | *CHRNA7* | *DOCK6* | *GCK* |
|  | *KCNQ1* | *NDUFAF1* | *PGM1* | *SDHA* | *AMT* | *CHRNB2* | *DOCK7* | *GCSH* | *KCNQ2* | *NDUFAF2* |
|  | *LGI1* | *NEDD4L* | *POMT1* | *SLC25A15* | *ATIC* | *COG6* | *EIF2B2* | *ADGRG1* | *LGR4* | *NEU1* |
|  | *POMT2* | *SLC25A19* | *ATN1* | *COG7* | *EIF2B3* | *ADGRV1* | *LIAS* | *NF1* | *PPOX* | *SLC25A22* |
|  | *ATP13A4* | *COG8* | *EIF2B4* | *GRIA3* | *LIG4* | *NGLY1* | *PPT1* | *SLC26A4* | *ATP1A2* | *COL18A1* |
|  | *EIF2B5* | *GRIN1* | *LMX1B* | *NHLRC1* | *PQBP1* | *SLC2A1* | *ATP1A3* | *COL4A1* | *ELP4* | *GRIN2A* |
|  | *LRPPRC* | *NHS* | *PRICKLE1* | *SLC35A1* | *ATP2A2* | *COMT* | *EMX2* | *GRIN2B* | *MAGI1* | *NID2* |
|  | *PRICKLE2* | *SLC35A2* | *ATP5A1* | *COQ2* | *EPB41L1* | *GSS* | *MAGI2* | *NOTCH3* | *PROC* | *SLC35C1* |
|  | *ATP6AP2* | *COQ9* | *EPHB2* | *GUSB* | *MAGT1* | *NPC1* | *PRODH* | *SLC46A1* | *ATP7A* | *COX14* |
|  | *EPM2A* | *GYS1* | *MAN1B1* | *NPC2* | *PRRT2* | *SLC6A8* | *ATPAF2* | *COX15* | *ERBB4* | *HAX1* |
|  | *MANBA* | *NPHP1* | *PSAP* | *SLC9A6* | *ATRX* | *COX6B1* | *ERLIN2* | *HDAC4* | *MAP2K1* | *NR3C1* |

**List of all covered genes in the thirteen TES panels**

| Name for TES Panels | Genes involved in the panel | | | | | | | | | | |
| --- | --- | --- | --- | --- | --- | --- | --- | --- | --- | --- | --- |
|  | *KRIT1* | | *NDUFS6* | *PNKD* | *SLC16A2* | *ARSB* | *CNTNAP2* | *ECM1* | *GNPTG* | *L2HGDH* | *NDUFS7* |
|  | *PNKP* | | *SLC17A5* | *ARSE* | *COA5* | *EEF1A2* | *GNS* | *LAMA2* | *NDUFS8* | *PNPO* | *SLC19A3* |
|  | *ARX* | | *COG1* | *EFHC1* | *GOSR2* | *LARGE1* | *NDUFV1* | *POLG* | *SLC1A3* | *ASAH1* | *COG4* |
|  | *PSAT1* | | *SLC9A9* | *ATXN10* | *CPA6* | *ETFA* | *HEXA* | *MAP2K2* | *NRAS* | *PTCH1* | *SMC1A* |
|  | *B4GALT1* | | *CPS1* | *ETFB* | *HEXB* | *MAPK10* | *NRXN1* | *PTPN11* | *SMPD1* | *BANK1* | *CPT1A* |
|  | *ETFDH* | | *HFE* | *MBD5* | *NTNG1* | *PTPN22* | *SMS* | *BCKDHA* | *CPT2* | *EVC* | *HGSNAT* |
|  | *MCCC2* | | *NUBPL* | *PUS1* | *SNIP1* | *BCKDHB* | *CSTB* | *FADD* | *HLA-DQA1* | *MCOLN1* | *OFD1* |
|  | *QDPR* | | *SNRPN* | *SOS1* | *CTNNA3* | *FASN* | *HLA-DQB1* | *MCPH1* | *OPA1* | *RAB39B* | *SOBP* |
|  | *SPAST* | | *SYNGAP1* | *TRPM6* | *ZDHHC15* | *SUCLA2* | *TMEM67* | *VPS13A* | *TUBGCP6* | *SPTAN1* | *SYNJ1* |
|  | *TSC1* | | *ZEB2* | *SUMF1* | *TMEM70* | *VPS13B* | *TUSC3* | *SPTLC2* | *SYP* | *TSC2* | *ZFYVE26* |
|  | *SUOX* | | *TNK2* | *VRK1* | *TYROBP* | *SRD5A3* | *SZT2* | *TSEN2* | *ZNF41* | *STXBP1* | *TMEM216* |
|  | *UBE3A* | | *ST3GAL5* | *SRPX2* | *TACO1* | *TSEN34* | *SURF1* | *TPP1* | *VRK2* | *TBX1* | *STRADA* |
|  | *ST3GAL2* | | *TBC1D24* | *TSEN54* | *SYN1* | *TREM2* | *WDR45* | *TCF4* | *STS* | *TBP* | *TUBA1A* |
|  | *SYN2* | | *TREX1* | *XK* | *TMEM165* |  |  |  |  |  |  |
| Hematological system diseases panel | |  |  |  |  |  |  |  |  |  |  |
|  | *HFE2* | | *MYC* | *RPL11* | *TBCE* | *ABCC6* | *CFH* | *FANCF* | *HK1* | *MYH9* | *RPL26* |
|  | *ABCA1* | | *CEBPA* | *FANCD2* | *HFE* | *MTTP* | *RIT1* | *TAZ* | *ABCB7* | *CETP* | *FANCE* |
|  | *TBXAS1* | | *ABCC8* | *CFHR1* | *FANCG* | *HMBS* | *NAGA* | *RPL35A* | *TERC* | *ABCG5* | *CFHR3* |
|  | *FANCI* | | *HPD* | *NBN* | *RPL5* | *TERT* | *ABCG8* | *CFI* | *FANCL* | *HRG* | *NF1* |
|  | *RPS10* | | *TET2* | *ADA* | *CFP* | *FANCM* | *IDH2* | *NFKBIA* | *RPS17L* | *TF* | *ADAMTS13* |
|  | *FAS* | | *IKBKG* | *NHP2* | *RPS19* | *THBD* | *AK1* | *CLDN19* | *FECH* | *IL6* | *NOP10* |

**List of all covered genes in the thirteen TES panels**

| Name for TES Panels | Genes involved in the panel | | | | | | | | | |
| --- | --- | --- | --- | --- | --- | --- | --- | --- | --- | --- |
|  | *RAD50* | *STAT3* | *CBL* | *F9* | *HBB* | *MMACHC* | *STIM1* | *CD36* | *FANCA* | *HBG1* |
|  | *RPS24* | *THPO* | *AK2* | *CPN1* | *FGA* | *IRF1* | *NOTCH3* | *RPS26* | *TINF2* | *ALAD* |
|  | *CREBBP* | *FLI1* | *ISCU* | *NPM1* | *RPS29* | *TMPRSS6* | *ALDH2* | *CSF3R* | *FLT3* | *ITGA2B* |
|  | *NR3C2* | *RTEL1* | *TNFRSF1A* | *ALDH4A1* | *CST3* | *FLT4* | *ITGB3* | *NRAS* | *RUNX1* | *TP53* |
|  | *ALDOA* | *CTC1* | *FOXC2* | *ITK* | *NT5C3* | *SBDS* | *TPI1* | *ALG12* | *CXCR4* | *FTL* |
|  | *ITPKC* | *ORAI1* | *SCN4A* | *TPP1* | *ANK1* | *CYB5R3* | *FXN* | *JAK2* | *OTC* | *SEC23B* |
|  | *TRPM6* | *AP3B1* | *CYCS* | *FXYD2* | *JAK3* | *P2RY12* | *SERPINC1* | *TUBB1* | *APOA1* | *TRPM6* |
|  | *DAPK1* | *G6PC3* | *KANSL1* | *PALB2* | *SERPINE1* | *UNC13D* | *APOA5* | *DCLRE1C* | *G6PD* | *KDM6A* |
|  | *PANK2* | *SERPINF2* | *USB1* | *APOB* | *DDX11* | *GATA1* | *KLF1* | *PCCA* | *SERPING1* | *VANGL2* |
|  | *ARG1* | *DKC1* | *GATA2* | *KLKB1* | *PCCB* | *SETBP1* | *VHL* | *ASXL1* | *EGF* | *GCLC* |
|  | *KNG1* | *PDE4D* | *SF3B1* | *VPS13B* | *ATM* | *EGLN1* | *GFI1* | *KRAS* | *PFKM* | *SH2D1A* |
|  | *VPS45* | *ATRX* | *ELANE* | *GGCX* | *KRIT1* | *PGK1* | *SLC11A2* | *VWDP* | *B2M* | *EPAS1* |
|  | *GK* | *LBR* | *PIEZO1* | *SLC19A2* | *VWF* | *B4GALT1* | *EPB41* | *GLRX5* | *LDLR* | *PIGA* |
|  | *SLC25A38* | *WAS* | *BCAM* | *EPB42* | *GNAS* | *LIG4* | *PKLR* | *SLC37A4* | *WRAP53* | *BIRC4* |
|  | *EPOR* | *GP1BA* | *LIPI* | *PLG* | *SLC40A1* | *WT1* | *BLM* | *ESCO2* | *GP1BB* | *LMAN1* |
|  | *PPOX* | *SLC4A1* | *XIAP* | *BMPR1A* | *ETV6* | *GP9* | *LMBRD1* | *PRF1* | *SLX4* | *YARS2* |
|  | *BRAF* | *EZH2* | *GPI* | *LPL* | *PROC* | *SMAD4* | *BRCA2* | *F11* | *GSR* | *LYST* |
|  | *PROS1* | *SMARCAL1* | *BRCC3* | *F12* | *GSS* | *MASTL* | *PTPN11* | *SMC1A* | *BRIP1* | *F13A1* |
|  | *HAMP* | *MCFD2* | *PUS1* | *SPTA1* | *BTK* | *F13B* | *HAX1* | *MECOM* | *RAB27A* | *SPTB* |
|  | *BUB1B* | *F5* | *HBA1* | *MEFV* | *RAD21* | *SRP72* | *CASP10* | *F8* | *HBA2* | *MLH1* |
|  | *MMADHC* | *RAG1* | *STX11* | *CDAN1* | *FANCB* | *HBG2* | *MPL* | *RAG2* | *STXBP2* | *CDH23* |

**List of all covered genes in the thirteen TES panels**

| Name for TES Panels | Genes involved in the panel | | | | | | | | | |
| --- | --- | --- | --- | --- | --- | --- | --- | --- | --- | --- |
|  | *FANCC* | *HCFC2* | *MSH2* | *RARA* | *TAT* | *CLDN16* | *RAD51C* |  |  |  |
| Dermatosis panel |  |  |  |  |  |  |  |  |  |  |
|  | *FBN1* | *FDPS* | *FECH* | *FERMT1* | *FGFR3* | *FH* | *FKBP14* | *FLG* | *FRMD7* | *FUCA1* |
|  | *FUS* | *GBA* | *GJA1* | *GJB2* | *GJB3* | *GJB4* | *GJB6* | *GLA* | *GLB1* | *GLI3* |
|  | *GNA11* | *GNAQ* | *GNAS* | *GNPAT* | *GORAB* | *GPR143* | *GRHL2* | *GSN* | *GTF2H5* | *HAMP* |
|  | *HARS* | *HCCS* | *HDAC8* | *HFE* | *HLCS* | *HOXA2* | *HOXC13* | *HPGD* | *HPS1* | *HPS3* |
|  | *HPS4* | *HPS5* | *HPS6* | *HR* | *HRAS* | *IFT122* | *IFT43* | *IGLL1* | *IL17F* | *IL17RA* |
|  | *IL1RN* | *IL2RG* | *IL31RA* | *IL36RN* | *IL7R* | *ING1* | *INSR* | *ITGA3* | *ITGA6* | *ITGB4* |
|  | *AAGAB* | *ABCA12* | *ABCB6* | *ABCC6* | *ABHD5* | *ACD* | *ACVRL1* | *ADA* | *ADAM10* | *ADAM17* |
|  | *ADAMTS17* | *ADAMTS2* | *ADAR* | *AGA* | *AGPS* | *AIRE* | *AKT1* | *ALAS2* | *ALDH18A1* | *ALDH3A2* |
|  | *ALOX12B* | *ALOXE3* | *ANOS1* |  | *AP1S1* | *AP1S3* | *AP3B1* | *APC* | *APCDD1* | *APOE* |
|  | *AQP5* | *ARHGAP31* | *ARHGAP6* | *ARPC3* | *ARSE* | *ASIP* | *ATF1* | *ATM* | *ATP2A2* | *ATP2C1* |
|  | *ATP6V0A2* | *ATP7A* | *AXIN2* | *B2M* | *B3GALT6* | *B4GALT7* | *BLM* | *BLNK* | *BLOC1S3* | *BLOC1S6* |
|  | *BMS1* | *BRAF* | *BTD* | *BTK* | *C10orf11* | *CARD14* | *CARD9* | *CAST* | *CBL* | *CD151* |
|  | *CD22* | *CD3D* | *CD79A* | *CD79B* | *CDH3* | *CDK4* | *CDKN2A* | *CDSN* | *CERS3* | *CFTR* |
|  | *CHRM3* | *CHRNA1* | *CHRND* | *CHRNG* | *CHST14* | *CHST8* | *CLCF1* | *CLDN1* | *CNTN2* | *COL14A1* |
|  | *COL17A1* | *COL1A1* | *COL1A2* | *COL3A1* | *COL5A1* | *COL5A2* | *COL5A3* | *COL7A1* | *COX7B* | *CREB1* |
|  | *CSTA* | *CTNNB1* | *CTSA* | *CTSC* | *CYLD* | *CYP26C1* | *CYP4F22* | *DCLRE1C* | *DDB2* | *DKC1* |
|  | *DLL4* | *DOCK6* | *DOCK8* | *DOCK9* | *DOLK* | *DSC2* | *DSC3* | *DSE* | *DSG1* | *DSG4* |
|  | *DSP* | *DST* | *DTNBP1* | *EBP* | *ECM1* | *EDA* | *EDA2R* | *EDAR* | *EDARADD* | *EDNRB* |
|  | *EFEMP2* | *ELN* | *ELOVL4* | *EMILIN2* | *ENG* | *ENPP1* | *EOGT* | *ERCC1* | *ERCC2* | *ERCC3* |

**List of all covered genes in the thirteen TES panels**

| Name for TES Panels | Genes involved in the panel | | | | | | | | | |
| --- | --- | --- | --- | --- | --- | --- | --- | --- | --- | --- |
|  | *ERCC4* | *ERCC5* | *ERCC6* | *ERCC8* | *EVC* | *EVC2* | *EWSR1* | *EXPH5* | *FAM111B* | *FBLN5* |
|  | *ITPKC* | *JAK3* | *JUP* | *KANK2* | *KCTD1* | *KIT* | *KITLG* | *KL* | *KLLN* | *KRAS* |
|  | *KRT1* | *KRT10* | *KRT13* | *KRT14* | *KRT16* | *KRT17* | *KRT2* | *KRT4* | *KRT5* | *KRT6A* |
|  | *KRT6B* | *KRT6C* | *KRT7* | *KRT71* | *KRT81* | *KRT83* | *KRT85* | *KRT86* | *KRT9* | *LAMA3* |
|  | *LAMB3* | *LAMC1* | *LAMC2* | *LCK* | *LIPH* | *LIPN* | *LMNA* | *LMX1B* | *LOR* | *LPAR6* |
|  | *LRRC8A* | *LTA* | *LTBP4* | *LYST* | *LZTR1* | *MANBA* | *MAP2K1* | *MAP2K2* | *MBTPS2* | *MC1R* |
|  | *MEN1* | *MIR184* | *MITF* | *MLH1* | *MLPH* | *MPLKIP* | *MSH2* | *MSH6* | *MSX1* | *MUTYH* |
|  | *MVD* | *MVK* | *MYH8* | *MYO5A* | *NAGA* | *NCSTN* | *NDUFB11* | *NECTIN1* | *NECTIN4* | *NEU1* |
|  | *NF1* | *NF2* | *NFKBIA* | *NHEJ1* | *NHP2* | *NIPAL4* | *NIPBL* | *NLGN4X* | *NLRC4* | *NLRP1* |
|  | *NLRP12* | *NLRP3* | *NOD2* | *NOP10* | *NOTCH1* | *NOTCH3* | *NPC1* | *NPC2* | *NRAS* | *NSDHL* |
|  | *OCA2* | *ORAI1* | *OSMR* | *PARN* | *PAX3* | *PDCD1* | *PDE11A* | *PDGFRA* | *PDGFRB* | *PEX7* |
|  | *PHGDH* | *PHYH* | *PIGL* | *PIK3CA* | *PIK3R1* | *PKP1* | *PLCG2* | *PLEC* | *PLOD1* | *PMS2* |
|  | *PMVK* | *PNPLA1* | *PNPLA2* | *POFUT1* | *POGLUT1* | *POLH* | *POMP* | *PORCN* | *PRKAR1A* | *PRKDC* |
|  | *PSAP* | *PSAT1* | *PSENEN* | *PSPH* | *PSTPIP1* | *PTCH1* | *PTCH2* | *PTEN* | *PTPN11* | *PTPN6* |
|  | *PTPRC* | *PYCR1* | *RAB27A* | *RAB27B* | *RAD21* | *RAF1* | *RAG1* | *RAG2* | *RBPJ* | *RECQL4* |
|  | *RET* | *RHBDF2* | *RIN2* | *RIT1* | *RMRP* | *RNASEH2A* | *RNASEH2B* | *RNASEH2C* | *RPL21* | *RSPO1* |
|  | *RTEL1* | *SAMD9* | *SAMHD1* | *SART3* | *SAT1* | *SCN9A* | *SDHB* | *SDHD* | *SERPINB7* | *SERPINB8* |
|  | *SHOC2* | *SLC11A1* | *SLC17A9* | *SLC24A5* | *SLC27A4* | *SLC29A3* | *SLC2A10* | *SLC39A13* | *SLC39A4* | *SLC45A2* |
|  | *SLC4A11* | *SLCO2A1* | *SLURP1* | *SMARCAD1* | *SMC1A* | *SMC3* | *SMPD1* | *SNAI2* | *SNAP29* | *SNRPE* |
|  | *SOS1* | *SOS2* | *SOX10* | *SPINK5* | *SPRED1* | *SRD5A3* | *SSH1* | *ST14* | *STAT1* | *STAT3* |
|  | *STIM1* | *STK11* | *STK4* | *STS* | *SUFU* | *SUMF1* | *TAT* | *TBXA2R* | *TEK* | *TERC* |
|  | *TERT* | *TFR2* | *TGFBI* | *TGM1* | *TGM5* | *TINF2* | *TLR1* | *TLR5* | *TMC6* | *TMC8* |

**List of all covered genes in the thirteen TES panels**

| Name for TES Panels | Genes involved in the panel | | | | | | | | | |
| --- | --- | --- | --- | --- | --- | --- | --- | --- | --- | --- |
|  | *TNFRSF10B* | *TNXB* | *TP53* | *TP63* | *TRAF3IP2* | *TRAF6* | *TREX1* | *TRPV3* | *TSC1* | *TSC2* |
|  | *TUBG1* | *TWIST2* | *TYR* | *TYRP1* | *UROD* | *UROS* | *USB1* | *VPS33B* | *VSX1* | *WDR19* |
|  | *WDR35* | *WNT10A* | *WRAP53* | *WRN* | *XPA* | *XPC* | *XYLT1* | *XYLT2* | *ZEB1* | *ZMPSTE24* |
|  | *ZNF750* | *RNF113A* |  |  |  |  |  |  |  |  |
| Immune panel |  |  |  |  |  |  |  |  |  |  |
|  | *ACP5* | *CD19* | *CYBA* | *IL1RN* | *NCF2* | *RNASEH2C* | *THBD* | *ACTB* | *CD247* | *CYBB* |
|  | *IL21R* | *NCF4* | *RNF168* | *TICAM1* | *ADA* | *CD27* | *DCLRE1C* | *IL2RA* | *NFKB2* | *RPSA* |
|  | *TINF2* | *ADAR* | *CD3D* | *DKC1* | *IL2RG* | *NFKBIA* | *RTEL1* | *TLR3* | *AICDA* | *CD3E* |
|  | *DNMT3B* | *IL36RN* | *NHP2* | *SAMHD1* | *TMC6* | *AIRE* | *CD3G* | *DOCK8* | *IL6* | *NLRP12* |
|  | *SBDS* | *TMC8* | *AK2* | *CD40* | *ELANE* | *IL7R* | *NLRP3* | *SEMA3E* | *TNFRSF13B* | *AP3B1* |
|  | *CD40LG* | *FADD* | *IRAK4* | *NOD2* | *SERPING1* | *TNFRSF13C* | *APOL1* | *CD46* | *FAS* | *IRF8* |
|  | *NOP10* | *SH2D1A* | *TNFRSF1A* | *ATM* | *CD59* | *FASLG* | *ISG15* | *NRAS* | *SH3BP2* | *TNFRSF4* |
|  | *BLM* | *CD79A* | *FCN3* | *ITCH* | *ORAI1* | *SLC29A3* | *TNFSF12* | *BLNK* | *CD79B* | *FERMT3* |
|  | *ITGB2* | *PIK3CD* | *SLC35C1* | *TRAF3* | *BTK* | *CD81* | *FOXN1* | *ITK* | *PIK3R1* | *SLC37A4* |
|  | *TRAF3IP2* | *C1QA* | *CD8A* | *FOXP3* | *JAK3* | *PLCG2* | *SLC46A1* | *TREX1* | *C1QB* | *CEBPE* |
|  | *FPR1* | *KRAS* | *PMS2* | *SMARCAL1* | *TTC7A* | *C1QC* | *CFB* | *G6PC3* | *LAMTOR2* | *PNP* |
|  | *SP110* | *TYK2* | *C1R* | *CFD* | *GATA2* | *LCK* | *POLE* | *SPINK5* | *UNC119* | *C1S* |
|  | *CFH* | *GFI1* | *LIG4* | *PRF1* | *STAT1* | *UNC13D* | *C2* | *CFHR1* | *HAX1* | *LPIN2* |
|  | *PRKCD* | *STAT2* | *UNC93B1* | *C3* | *CFHR2* | *ICOS* | *LRBA* | *PRKDC* | *STAT3* | *UNG* |
|  | *C4A* | *CFHR3* | *IFNG* | *LYST* | *PSMB8* | *STAT5B* | *USB1* | *C4B* | *CFHR4* | *IFNGR1* |
|  | *MAGT1* | *PSTPIP1* | *STIM1* | *VPS13B* | *C5* | *CFHR5* | *IFNGR2* | *MALT1* | *PTPRC* | *STK4* |

**List of all covered genes in the thirteen TES panels**

| Name for TES Panels | Genes involved in the panel | | | | | | | | | |
| --- | --- | --- | --- | --- | --- | --- | --- | --- | --- | --- |
|  | *VPS45* | *C6* | *CFI* | *IGLL1* | *MASP1* | *RAB27A* | *STX11* | *WAS* | *C7* | *CFP* |
|  | *IKBKB* | *MASP2* | *RAC2* | *STXBP2* | *WIPF1* | *C8A* | *CHD7* | *IKBKG* | *MCM4* | *RAG1* |
|  | *TAP1* | *XIAP* | *C8B* | *CIITA* | *IKZF1* | *MEFV* | *RAG2* | *TAP2* | *ZAP70* | *C8G* |
|  | *CLEC7A* | *IL10* | *MRE11A* | *RBCK1* | *TAPBP* | *ZBTB24* | *C9* | *COLEC11* | *IL10RA* | *MS4A1* |
|  | *RFX5* | *TAZ* | *CARD11* | *CORO1A* | *IL10RB* | *MTHFD1* | *RFXANK* | *TBK1* | *CARD14* | *CR2* |
|  | *IL12B* | *MVK* | *RFXAP* | *TBX1* | *CARD9* | *CSF2RA* | *IL12RB1* | *MYD88* | *RHOH* | *TCF3* |
|  | *CASP10* | *CTSC* | *IL17F* | *NBN* | *RNASEH2A* | *TCN2* | *CASP8* | *CXCR4* | *IL17RA* | *NCF1* |
|  | *RNASEH2B* | *TERT* |  |  |  |  |  |  |  |  |
| Nephrosis panel |  |  |  |  |  |  |  |  |  |  |
|  | *AQP2* | *CLCN5* | *DMP1* | *KCNJ10* | *SCNN1B* | *WNK1* | *ATP6V0A4* | *EGF* | *KLHL3* | *SCNN1G* |
|  | *SLC4A1* | *WNK4* | *ATP6V1B1* | *ENPP1* | *MUC1* | *SLC12A1* | *SLC4A4* | *AVPR2* | *CLDN19* | *FGF23* |
|  | *OCRL* | *SLC12A3* | *SLC5A2* | *BSND* | *CNNM2* | *FXYD2* | *PHEX* | *SLC1A1* | *SLC7A7* | *CA2* |
|  | *CTNS* | *KCNA1* | *REN* | *SLC22A12* | *TRPM6* | *CASR* | *CUL3* | *KCNJ1* | *SCNN1A* | *SLC2A2* |
|  | *UMOD* | *SLC34A1* | *CLCNKB* | *CLDN16* |  |  |  |  |  |  |
| Ophthalmic disease panel |  |  |  |  |  |  |  |  |  |  |
|  | *ABCA4* | *CACNA1F* | *FLVCR1* | *KIF7* | *OPA6* | *PRPH2* | *SDCCAG8* | *TRPM1* | *ABCB6* | *CACNA2D4* |
|  | *FSCN2* | *KLHL7* | *OPN1LW* | *RAB28* | *SEMA4A* | *TSPAN12* | *ABHD12* | *CC2D2A* | *FZD4* | *LCA5* |
|  | *OPN1MW* | *RAX2* | *SHH* | *TTC8* | *ADAM9* | *CCDC28B* | *GDF3* | *LRAT* | *PAX6* | *RBP3* |
|  | *SLC24A1* | *TTLL5* | *AHI1* | *CDH23* | *GDF6* | *LRIT3* | *PCDH15* | *RD3* | *SLC25A4* | *TUBA8* |
|  | *AIPL1* | *CDHR1* | *GNAT1* | *LRP5* | *PCYT1A* | *RDH12* | *SLC38A8* | *TULP1* | *ARL13B* | *CEP290* |
|  | *GP1BA* | *LZTFL1* | *PDE6A* | *RDH5* | *SLC7A14* | *UCHL1* | *ARL2BP* | *CEP41* | *GPR179* | *MAK* |

**List of all covered genes in the thirteen TES panels**

| Name for TES Panels | Genes involved in the panel | | | | | | | | | |
| --- | --- | --- | --- | --- | --- | --- | --- | --- | --- | --- |
|  | *TCTN3* | *WFS1* | *BBS4* | *CNNM4* | *HARS* | *NEK2* | *PLK1S1* | *RP9* | *TENM3* | *YAP1* |
|  | *BBS5* | *CRB1* | *IDH3B* | *NMNAT1* | *POC1B* | *RPE65* | *TIMM8A* | *ZNF408* | *BBS7* | *CRX* |
|  | *IFT27* | *NPHP1* | *POLG* | *RPGR* | *TIMP3* | *ZNF423* | *BBS9* | *CSPP1* | *IKBKG* | *NPHP4* |
|  | *POLG2* | *RPGRIP1* | *TMEM126A* | *ZNF513* | *BEST1* | *CYP4V2* | *IMPDH1* | *NR2E3* | *PRCD* | *RPGRIP1L* |
|  | *TMEM138* | *C10orf2* | *DFNB31* | *IMPG2* | *NR2F1* | *PROM1* | *RQCD1* | *TMEM216* | *C2orf71* | *DHDDS* |
|  | *INPP5E* | *NRL* | *PRPF3* | *RRM2B* | *TMEM231* | *C5orf42* | *DNA2* | *IQCB1* | *NYX* | *PRPF31* |
|  | *PDE6B* | *RGR* | *SNRNP200* | *UNC119* | *ARL6* | *CERKL* | *GPR98* | *MERTK* | *PDE6C* | *RHO* |
|  | *SOX2* | *USH1C* | *BBIP1* | *CIB2* | *GRK1* | *MKKS* | *PDE6D* | *RIMS1* | *SPATA7* | *USH1G* |
|  | *BBS1* | *CISD2* | *GRM6* | *MKS1* | *PDE6G* | *RLBP1* | *STRA6* | *USH2A* | *BBS10* | *CLRN1* |
|  | *GUCA1A* | *MYO7A* | *PDE6H* | *ROM1* | *TBX1* | *VSX2* | *BBS12* | *CNGA1* | *GUCA1B* | *NBAS* |
|  | *PDZD7* | *RP1* | *TCTN1* | *WDPCP* | *BBS2* | *CNGB1* | *GUCY2D* | *NDP* | *PITPNM3* | *RP2* |
|  | *RS1* | *TMEM237* | *C8orf37* | *ELOVL4* | *KCNJ13* | *OFD1* | *PRPF4* | *RYR1* | *TMEM67* | *CA4* |
|  | *EYS* | *KCNV2* | *OPA1* | *PRPF6* | *SAG* | *TOPORS* | *CABP4* | *FAM161A* | *KIF11* | *OPA3* |
|  | *PRPF8* | *SALL2* | *TRIM32* |  |  |  |  |  |  |  |
| Cardiovascular system panel |  |  |  |  |  |  |  |  |  |  |
|  | *AARS2* | *CACNA1C* | *DNAJC19* | *GHR* | *KMT2D* | *NEBL* | *PUF60* | *SURF1* | *ABCA1* | *CACNA1D* |
|  | *DOCK6* | *GHRL* | *KRAS* | *NEK1* | *RAB23* | *SYNE1* | *ABCA3* | *CACNA1F* | *DOLK* | *GJA1* |
|  | *KRT18* | *NEU1* | *RAD21* | *SYNE2* | *ABCA4* | *CACNA1H* | *DPM1* | *GJA5* | *KRT8* | *NEXN* |
|  | *RAD51C* | *SYNM* | *ABCC6* | *CACNA1S* | *DPM3* | *GJC2* | *LAMA2* | *NF1* | *RAF1* | *TAB2* |
|  | *ABCC9* | *CACNA2D1* | *DPP6* | *GLA* | *LAMA4* | *NFU1* | *RAI1* | *TACO1* | *ABCD4* | *CACNA2D4* |

**List of all covered genes in the thirteen TES panels**

| Name for TES Panels | Genes involved in the panel | | | | | | | | | |
| --- | --- | --- | --- | --- | --- | --- | --- | --- | --- | --- |
|  | *FADD* | *HPGD* | *MIAT* | *PEX2* | *SCNN1B* | *TRIM37* | *ANGPTL4* | *COL1A1* | *FAH* | *HPS1* |
|  | *MIB1* | *PEX5* | *SCNN1G* | *TRIM63* | *ANK2* | *COL1A2* | *FAM114A2* | *HPS4* | *MID1* | *PEX6* |
|  | *SCO2* | *TRMU* | *ANKRD1* | *COL3A1* | *FAM167A* | *HRAS* | *MKKS* | *PEX7* | *SDCCAG8* | *TRNT1* |
|  | *FLNC* | *ITGA2* | *MYH7B* | *PNPLA3* | *SLC22A5* | *WDPCP* | *B3GALNT2* | *CSF2RA* | *FLT1* | *ITGB3* |
|  | *MYH8* | *POLD1* | *SLC25A20* | *WDR35* | *B3GALT6* | *CSRP3* | *FMO3* | *ITM2B* | *MYH9* | *POLG* |
|  | *DSC2* | *GLB1* | *LAMP2* | *NIPBL* | *RANGRF* | *TALDO1* | *ABCG5* | *CACNB2* | *DSE* | *GLI3* |
|  | *LARGE1* | *NKX2-5* | *RASA1* | *TAZ* | *ABCG8* | *CALM1* | *DSG2* | *GLMN* | *LBR* | *NKX2-6* |
|  | *RBCK1* | *TBX1* | *ABO* | *CALM2* | *DSP* | *GMPPB* | *LCAT* | *NOD2* | *RBM10* | *TBX20* |
|  | *ACACB* | *CALM3* | *DST* | *GNA11* | *LDB3* | *NODAL* | *RBM20* | *TBX5* | *ACAD8* | *CALR3* |
|  | *DTNA* | *GNA12* | *LDLR* | *NOS1AP* | *RBM8A* | *TCAP* | *ACAD9* | *CASP3* | *DVL1* | *GNAI2* |
|  | *LDLRAP1* | *NOS2* | *RBPJ* | *TCF21* | *ACADM* | *CASQ1* | *DYNC2H1* | *GNAS* | *LEFTY2* | *NOS3* |
|  | *RECQL4* | *TCTN3* | *ACADS* | *CASQ2* | *DYRK1B* | *GNB3* | *LGALS2* | *NOTCH1* | *RET* | *TERT* |
|  | *ACADVL* | *CASR* | *ECE1* | *GNPTAB* | *LIAS* | *NOTCH2* | *RGS5* | *TET2* | *ACE* | *CAV1* |
|  | *EEPD1* | *GNPTG* | *LIPA* | *NOTCH3* | *RIT1* | *TFAP2B* | *ACTA1* | *CAV3* | *EFEMP2* | *GPAM* |
|  | *LIPC* | *NPC1* | *RPL11* | *TGFB2* | *ACTA2* | *CBL* | *EGLN1* | *GPC3* | *LIPG* | *NPC2* |
|  | *RPL15* | *TGFB3* | *ACTB* | *CBS* | *EGLN2* | *GPD1* | *LIPI* | *NPHP3* | *RPL26* | *TGFBR1* |
|  | *ACTC1* | *CCBE1* | *EHBP1* | *GPD1L* | *LMF1* | *NPPA* | *RPL35A* | *TGFBR2* | *ACTG1* | *CCND1* |
|  | *EHMT1* | *GPIHBP1* | *LMNA* | *NR2F2* | *RPL5* | *TGFBR3* | *ACTN2* | *CD163* | *EIF2AK4* | *GPX4* |
|  | *LPA* | *NR3C1* | *RPS10* | *THBD* | *ACVR1C* | *CD36* | *EIF4B* | *GRIP1* | *LPIN1* | *NR3C2* |
|  | *RPS17* | *THBS1* | *ACVR2B* | *CD40* | *ELAC2* | *GRN* | *LPL* | *NRAS* | *RPS19* | *THOC6* |
|  | *SLC25A3* | *WDR60* | *B3GAT3* | *CST3* | *FN1* | *ITPKC* | *MYL2* | *POLG2* | *SLC25A4* | *WFS1* |

**List of all covered genes in the thirteen TES panels**

| Name for TES Panels | Genes involved in the panel | | | | | | | | | |
| --- | --- | --- | --- | --- | --- | --- | --- | --- | --- | --- |
|  | *ACVRL1* | *CD40LG* | *ELN* | *GSN* | *LRP5* | *NSD1* | *RPS24* | *TIMD4* | *ADA2* | *CD96* |
|  | *ELOVL4* | *GTPBP3* | *LRP6* | *NSDHL* | *RPS26* | *TIRAP* | *ADAMTSL2* | *CDC73* | *EMD* | *GUCY1A3* |
|  | *LRP8* | *NUBPL* | *RPS28* | *TJP2* | *ADD1* | *CDKN1B* | *ENG* | *GUSB* | *LTA* | *NUP155* |
|  | *RPS29* | *TLL1* | *ADRB1* | *CDKN1C* | *EOGT* | *GYG1* | *LTBP2* | *OBSCN* | *RPS6KA3* | *TLR4* |
|  | *ADRB2* | *CDKN2A* | *EPAS1* | *GYS1* | *LYRM7* | *OCRL* | *RPS7* | *TM6SF2* | *AFF4* | *CEP120* |
|  | *EPG5* | *H19* | *LYZ* | *OFD1* | *RPSA* | *TMEM126A* | *AGA* | *CEP19* | *EPHX2* | *HADH* |
|  | *LZTFL1* | *OLR1* | *RRM2B* | *TMEM127* | *AGK* | *CEP290* | *EPOR* | *HADHA* | *LZTR1* | *PALB2* |
|  | *RYR1* | *TMEM43* | *AGL* | *CERS1* | *ERCC1* | *HADHB* | *MAMLD1* | *PAX6* | *RYR2* | *TMEM5* |
|  | *AGPAT2* | *CETP* | *ERCC4* | *HAMP* | *MAP2K1* | *PCCA* | *S100A12* | *TMEM70* | *AGT* | *CFC1* |
|  | *ERCC6* | *HBB* | *MAP2K2* | *PCCB* | *SACS* | *TMPO* | *AGTR1* | *CFH* | *ESCO2* | *HCCS* |
|  | *MAT2A* | *PCSK7* | *SALL1* | *TNFSF4* | *AGXT* | *CFI* | *ETFA* | *HCN4* | *MAX* | *PCSK9* |
|  | *SALL4* | *TNNC1* | *AIP* | *CFTR* | *ETFB* | *HDAC8* | *MC4R* | *PDE11A* | *SARS2* | *TNNI3* |
|  | *AKAP10* | *CHD7* | *ETFDH* | *HEXA* | *MCOLN1* | *PDE3A* | *SBDS* | *TNNI3K* | *AKAP9* | *CHKB* |
|  | *EVC* | *HEXB* | *MCU* | *PDE8B* | *SCN10A* | *TNNT2* | *AKT1* | *CHRM2* | *EVC2* | *HEY2* |
|  | *MED13L* | *PDGFC* | *SCN1A* | *TNNT3* | *AKT3* | *CHST14* | *EVI5* | *HFE* | *MED25* | *PDGFRA* |
|  | *SCN1B* | *TNXB* | *ALB* | *CHST3* | *EYA1* | *HFE2* | *MEF2A* | *PDHA1* | *SCN2B* | *TOPBP1* |
|  | *ALDH1A2* | *CISD2* | *EYA4* | *HLA-A* | *MEFV* | *PDLIM3* | *SCN3B* | *TP63* | *ALDH2* | *CITED2* |
|  | *F12* | *HLA-B* | *MEGF8* | *PDSS1* | *SCN4A* | *TPM1* | *ALG1* | *CLCNKB* | *F13A1* | *HMBS* |
|  | *MEN1* | *PDSS2* | *SCN4B* | *TPM2* | *ALG10* | *COA5* | *F2* | *HMGCR* | *MFAP5* | *PET100* |
|  | *SCN5A* | *TRDN* | *ALMS1* | *COA6* | *F7* | *HNF1A* | *MGAT1* | *PEX1* | *SCN9A* | *TRIB1* |
|  | *AMPD1* | *COG7* | *F9* | *HOXA1* | *MGME1* | *PEX16* | *SCNN1A* | *TRIM32* | *ANGPTL3* | *COL18A1* |

**List of all covered genes in the thirteen TES panels**

| Name for TES Panels | Genes involved in the panel | | | | | | | | | |
| --- | --- | --- | --- | --- | --- | --- | --- | --- | --- | --- |
|  | *ANKS6* | *COL4A1* | *FANCA* | *HRG* | *MKS1* | *PGM1* | *SDHA* | *TRPA1* | *AP1S1* | *COL4A2* |
|  | *FANCC* | *HSD11B2* | *MLXIPL* | *PHKA2* | *SDHAF1* | *TRPM4* | *APOA1* | *COL4A3* | *FANCD2* | *HSD17B10* |
|  | *MLYCD* | *PHKG2* | *SDHAF2* | *TSC1* | *APOA2* | *COL5A1* | *FANCE* | *HTR2B* | *MMAB* | *PHYH* |
|  | *SDHAF3* | *TSFM* | *APOA4* | *COL5A2* | *FANCI* | *HTRA1* | *MMACHC* | *PIEZO2* | *SDHB* | *TSPYL1* |
|  | *APOA5* | *COL6A1* | *FASTKD2* | *HYLS1* | *MPI* | *PIGA* | *SDHC* | *TSR2* | *APOB* | *COL6A2* |
|  | *FAT4* | *HYT1* | *MPL* | *PIGL* | *SDHD* | *TTC19* | *APOC2* | *COL6A3* | *FBLN2* | *HYT2* |
|  | *MRE11* | *PIGM* | *SELE* | *TTC8* | *APOC3* | *COQ2* | *FBN1* | *HYT3* | *MRPL3* | *PIGN* |
|  | *SEMA3A* | *TTN* | *APOE* | *COQ4* | *FBN2* | *HYT4* | *MRPL44* | *PIGO* | *SEMA3E* | *TTPA* |
|  | *APOPT1* | *COQ9* | *FCGR2A* | *HYT5* | *MRPS16* | *PIGT* | *SERPINA6* | *TTR* | *APP* | *CORIN* |
|  | *FGA* | *HYT6* | *MRPS22* | *PIK3CA* | *SERPINC1* | *TWIST1* | *ARHGAP31* | *COX10* | *FGD1* | *IDH2* |
|  | *MTHFR* | *PITX2* | *SERPIND1* | *TXNL4A* | *ARL6* | *COX14* | *FGF12* | *IDS* | *MTM1* | *PKD1* |
|  | *SFTPA2* | *TXNRD2* | *ARMC5* | *COX15* | *FGFR1* | *IFT27* | *MTO1* | *PKD1L3* | *SFTPC* | *UQCC2* |
|  | *ARSB* | *COX20* | *FGFR2* | *IFT43* | *MTTP* | *PKP2* | *SFTPD* | *UQCC3* | *ARX* | *COX6B1* |
|  | *FGFR3* | *IGBP1* | *MUC1* | *PKP4* | *SGCD* | *UQCRB* | *ASAH1* | *COX7B* | *FH* | *IGHMBP2* |
|  | *MUC5B* | *PLA2G7* | *SGCG* | *UQCRC2* | *ASCC3* | *CPOX* | *FHL1* | *IKBKAP* | *MUT* | *PLAT* |
|  | *SH2B3* | *UQCRQ* | *ATM* | *CPS1* | *FHL2* | *IKBKG* | *MVK* | *PLCE1* | *SHOC2* | *USB1* |
|  | *ATP1B1* | *CPT1A* | *FHOD3* | *IL10* | *MYBPC3* | *PLIN1* | *SKI* | *USF1* | *ATP2A2* | *CPT2* |
|  | *FIG4* | *IL23R* | *MYCN* | *PLN* | *SLC12A1* | *VCL* | *ATP5A1* | *CREBBP* | *FKRP* | *ILK* |
|  | *MYF6* | *PLOD1* | *SLC12A3* | *VCP* | *ATP5E* | *CRELD1* | *FKTN* | *ISCU* | *MYH11* | *PMFBP1* |
|  | *SLC12A4* | *VHL* | *ATP6V1B2* | *CRELD2* | *FLNA* | *ISL1* | *MYH6* | *PMM2* | *SLC17A5* | *VKORC1* |
|  | *ATPAF2* | *CRKL* | *FLNB* | *ISPD* | *MYH7* | *PNPLA2* | *SLC17A8* | *VPS13B* | *ATRX* | *CRYAB* |

**List of all covered genes in the thirteen TES panels**

| Name for TES Panels | Genes involved in the panel | | | | | | | | | |
| --- | --- | --- | --- | --- | --- | --- | --- | --- | --- | --- |
|  | *B4GALT1* | *CTC1* | *FOXC1* | *IVD* | *MYL3* | *POMGNT1* | *WNK1* | *B4GAT1* | *CTF1* | *FOXC2* |
|  | *JAG1* | *MYLK* | *POMGNT2* | *WNK4* | *BAG3* | *CTNNA3* | *FOXF1* | *JAK2* | *MYLK2* | *POMK* |
|  | *SLC37A4* | *WNT3* | *BANF1* | *CTSA* | *FOXH1* | *JPH2* | *MYO6* | *POMT1* | *SLC6A4* | *WT1* |
|  | *BAZ1B* | *CUL3* | *FOXRED1* | *JUP* | *MYOM1* | *POMT2* | *SLMAP* | *XK* | *BBIP1* | *CX3CR1* |
|  | *FRAS1* | *KANSL1* | *MYOT* | *PORCN* | *SMAD3* | *XPNPEP3* | *BBS1* | *CYC1* | *FREM2* | *KAT6B* |
|  | *MYOZ1* | *PPARA* | *SMAD4* | *ZEB2* | *BBS10* | *CYP11A1* | *FRZB* | *KCNA1* | *MYOZ2* | *PPARG* |
|  | *SMAD6* | *ZFHX3* | *BBS12* | *CYP11B1* | *FSCN2* | *KCNA5* | *MYPN* | *PPOX* | *SMAD9* | *ZFPM2* |
|  | *BBS2* | *CYP17A1* | *FTO* | *KCND3* | *NAA10* | *PPP1R17* | *SMC1A* | *ZIC3* | *BBS4* | *CYP1B1* |
|  | *FUCA1* | *KCNE1* | *NAGA* | *PQBP1* | *SMC3* | *ZMPSTE24* | *BBS5* | *CYP21A2* | *FXN* | *KCNE2* |
|  | *NDUFA1* | *PRDM16* | *SMPD1* | *ZNF469* | *BBS7* | *CYP24A1* | *G6PC* | *KCNE3* | *NDUFA11* | *PRDM6* |
|  | *SNTA1* | *BBS9* | *CYP27A1* | *G6PC3* | *KCNE5* | *NDUFAF1* | *PRG4* | *SORT1* | *BCOR* | *CYP3A5* |
|  | *GAA* | *KCNH1* | *NDUFAF2* | *PRKAG2* | *SOS1* | *BCS1L* | *D2HGDH* | *GATA1* | *KCNH2* | *NDUFAF3* |
|  | *SOS2* | *BEST1* | *DAG1* | *GATA4* | *KCNJ1* | *NDUFAF4* | *PRKG1* | *SOX2* | *BIN1* | *DBH* |
|  | *GATA5* | *KCNJ2* | *NDUFAF5* | *PROC* | *SOX7* | *BLK* | *DCAF8* | *GATA6* | *KCNJ5* | *NDUFB11* |
|  | *PROS1* | *SP110* | *BLM* | *DCHS1* | *GATAD1* | *KCNJ6* | *NDUFB3* | *PRRX1* | *SP3* | *BMPR2* |
|  | *DES* | *GBA* | *KCNJ8* | *NDUFB9* | *PRSS1* | *SPECC1L* | *BOLA3* | *DHCR24* | *GBE1* | *KCNK18* |
|  | *NDUFS1* | *PSEN1* | *SPEG* | *BRAF* | *DHCR7* | *GCDH* | *KCNK3* | *NDUFS2* | *PSEN2* | *SPINK1* |
|  | *BSCL2* | *DIS3L2* | *GCKR* | *KCNMB1* | *NDUFS3* | *PSMA6* | *SPRED1* | *BVES* | *DLL3* | *GCLC* |
|  | *KCNQ1* | *NDUFS4* | *PSMB8* | *SRI* | *C2* | *DLL4* | *GCLM* | *KCNQ1OT1* | *NDUFS6* | *PTEN* |
|  | *STAMBP* | *C4A* | *DMD* | *GDF1* | *KCTD7* | *NDUFS7* | *PTF1A* | *STAT4* | *C6orf183* | *DNAH11* |

**List of all covered genes in the thirteen TES panels**

| Name for TES Panels | Genes involved in the panel | | | | | | | | | |
| --- | --- | --- | --- | --- | --- | --- | --- | --- | --- | --- |
|  | *PTPN11* | *STOX1* | *CACNA1B* | *DNAI1* | *GFM1* | *KLHL3* | *NDUFV2* | *PTRF* | *SUMO4* | *SLC29A3* |
|  | *GDF2* | *KIF1B* | *NDUFS8* | *PTGIS* | *STK4* | *CACNA1A* | *DNAH5* | *GDNF* | *KLF10* | *NDUFV1* |
|  | *SLC2A10* | *PRKAR1A* |  |  |  |  |  |  |  |  |
| Mitochondrial gene panel |  |  |  |  |  |  |  |  |  |  |
|  | *MT-ND1* | *MT-ND2* | *MT-ND3* | *MT-ND4* | *MT-ND4L* | *MT-ND5* | *MT-ND6* | *MT-Cytb* | *MT-COX1* | *MT-COX2* |
|  | *MT-COX3* | *MT-ATP6* | *MT-ATP8* | *MT-TA* | *MT-TR* | *MT-TN* | *MT-TD* | *MT-TC* | *MT-TT* | *MT-TE* |
|  | *MT-TQ* | *MT-TG* | *MT-TH* | *MT-TI* | *MT-TK* | *MT-TM* | *MT-TF* | *MT-TP* | *MT-TW* | *MT-TY* |
|  | *MT-TV* | *MT-TL1* | *MT-TL2* | *MT-TS1* | *MT-TS2* | *MT-RNR1* | *MT-RNR2* |  |  |  |

Abbreviation: TES, Targeted Exome Sequencing; DMD, Duchenne muscular dystrophies; SMA, Spinal Muscular trophy.

**Specific Phenotypes Associated with a Positive Diagnosis.**

In this study, we selected 22 phenotypes of likely separated genetic etiology. These phenotypes occur most frequently. They was selected based on extensive literature research and the following criteria: i) they potentially discriminate between healthy subjects and patients; and ii) some specific biomarkers can be measured in the blood or other samples collected according to generally accepted and applicable procedures for bio sample collection. Fourteen different phenotypes fell into 7 root HPO term ‘Phenotypic abnormality’ and the remaining four were separate HPO terms (Supplementary Table 1). Many infants had more than one phenotype within the same top-branch HPO category, but each applicable top-branch category was only tallied once per infant.

Odds Ratio (OR) was calculated to verify each top-level branching of HPO categories and their composition in predicting a molecular diagnosis. A multiple-factor logistic regression was used to analyze initially the HPO category compositions followed by the calculation of OR of each composition’s predicting value.

**Independent Predictors of a Positive Diagnosis.**

To identify independent predictors of a positive diagnosis, we started with a full model including all variables (7 root HPO term, 22 individual phenotypes, their compositions, the number of phenotypes (reflected by the HPO term number) involved in every patient). A multivariable logistic regression analysis was performed to evaluate the independent significance.

**The Diagnostic Performance of the Independent Predictors in Identifying the Patients with a Positive Diagnosis in NICU.**

We analyzed receiver operating characteristic (ROC) curves according to the values of sensitivity, specificity, and Youden’s index. We defined areas under the ROC curves (AUCs) < 0.7 as having poor discriminatory value, 0.7-0.8 as minimally accurate, 0.8-0.9 as having good accuracy, and > 0.9 as having excellent accuracy. For those variables with a Youden’s index >0.7, we determined their optimal cutoff value that differentiated patients with a positive diagnosis and those without.

**Supplementary Tables**

**Table S1. Profiles of 142 patients molecularly diagnosed in three NICUs.**

| ID | LOVD  Individual ID | Gene(s) | Variants | Variant class (LP/P/VUS) | Disease(s) | Inheritance  pattern | Segregation | Zygosity | Molecular diagnostic technology |
| --- | --- | --- | --- | --- | --- | --- | --- | --- | --- |
| 1 | 00305888 | *ABCC8* (NM_000352.6) | c.1671+2T>C | LP | Familial hyperinsulinemic hypoglycemia [MIM:256450] | AD | Inherited  (from father) | het | TES panel |
| 2 | 00303391 | *TGM1* (NM_000359.3) | c.1130G>A;  c.871G>A | LP  P | Ichthyosis [MIM:242300] | AR | Inherited (from father + mother) | het | TES panel |
| 3 | 00305889 | *JAG1* (NM_000214.3) | c.2922dupT | P | Alagille syndrome 1 [MIM:118450] | AD | De novo | het | TES panel |
| 4 | 00305890 | *DMD* (NM_004006.2) | c.8713C>T | P | Duchenne muscular dystrophies [MIM:310200] | XR | Inherited  (from mother) | hemi | TES panel |
| 5 | 00305891 | *KIF11* (NM_004523.4) | c.77+1G>A | P | Microcephaly with or without chorioretinopathy, lymphedema, or mental retardation [MIM:152950] | AD | De novo | het | TES panel |
| 6 | 00306817 | *FLI1* (NM_002017.5) | FLI1 heterozygous del | P | Bleeding disorder, platelet-type, 21 [MIM:617443] | AD | De novo | het | TES panel |
| 7 | 00305892 | *ITGB2* (NM_000211.5) | c.817G>A; 817G>A | P  P | Leukocyte adhesion deficiency [MIM:116920] | AR | De novo/Inherited  (from father) | hom | TES panel |
| 8 | 00305893 | *EPHB4* (NM_004444.5) | c.2354G>A | P | Lymphatic malformation 7 [MIM:617300] | AD | De novo | het | WES |
| 9 | 00305894 | *OTC* (NM_000531.6) | c.119G>A | LP | Ornithine transcarbamylase deficiency [MIM:311250] | XR | Inherited  (from mother) | hemi | TES panel |
| 10 | 00305895 | *AVPR2* (NM_000054.6) | c.963C>A | P | X-linked nephrogenic diabetes insipidu [MIM:304800] | XR | Inherited  (from mother) | hemi | WES |
| 11 | 00305896 | *ALPL* (NM_000478.6) | c.18delA;  c.1101_1103delCTC | P  P | Hypophosphatasia, infantile forms [MIM:241500] | AR | Inherited (from father + mother) | het | WES |
| 12 | 00305897 | 16p11.2-p12.2 del | Chr 16p11.2-p12.2 del | LP | Chromosome 16p12.2-p11.2 deletion syndrome [MIM:613604] | Isolated cases | Unkown  (only proband) | het | WES |
| 13 | 00305898 | *DMD* (NM_004006.2) | Ex 8-9 del (c.650-?_960 + ?del  (out of frame) | P | Duchenne muscular dystrophies [MIM:310200] | XR | Inherited  (from mother) | hemi | TES panel |
| 14 | 00305899 | *DMD* (NM_004006.2) | Ex 8-9 del  (c.650-?_960 + ?del  (out of frame) | P | Duchenne muscular dystrophies [MIM:310200] | XR | Inherited  (from mother) | hemi | TES panel |

Table S1 Continued

| ID | LOVD  Individual ID | Gene(s) | Variants | Variant class (LP/P/VUS) | Disease(s) | Inheritance  pattern | Segregation | Zygosity | Molecular diagnostic technology |
| --- | --- | --- | --- | --- | --- | --- | --- | --- | --- |
| 15 | 00305900 | *MMACHC* (NM_015506.3) | c.217C>T;  c.609G>A | P  P | Methylmalonic aciduria and homocystinuria, cblC type [MIM:277400] | AR | Inherited (from father + mother) | het | TES panel |
| 16 | 00305901 | *ATP6V0A4* (NM_020632.3) | c.639+1G>A; 639+1G>A | P | Renal tubular acidosis, distal, autosomal recessive [MIM:602722] | AR | Inherited (from father + mother) | hom | TES panel |
| 17 | 00305902 | *ABCC8* (NM_000352.3) | c.2348T>G;  c.3124_3126delACCinsCAGCCAGGAACTG | LP  LP | Hyperinsulinemic hypoglycemia, familial, 1[MIM:256450] | AR | Inherited (from father + mother) | het | WES |
| 18 | 00305903 | *ABCC8* (NM_000352.3) | c.4412-13G>A;  c.2992C>T | LP  P | Hypoglycaemia, persistent hyperinsulinaemic [MIM:256450] | AR | Inherited (from father + mother) | het | TES panel |
| 19 | 00305904 | *CPS1* (NM_001875.4) | c.2162G>A;  c.2938G>A | P  LP | Carbamoylphosphate synthetase I deficiency [MIM:237300] | AR | Inherited (from father + mother) | het | WES |
| 20 | 00305905 | *HBG2* (NM_000184.3) | c.190C> T | LP | Fetal hemoglobin quantitative trait locus1 [MIM:141749] | AD | Unkown  (only proband) | het | TES panel |
| 21 | 00305906 | *MMACHC* (NM_015506.3) | c.394C>T;  c.609G>A | P  P | Methylmalonic aciduria and homocystinuria, cblC type [MIM:277400] | AR | Inherited (from father + mother) | het | TES panel |
| 22 | 00306833 | 22q11.2 del | Chr 22q11.2 del | LP | DiGeorge Syndrom [MIM:188400] | AD | Unkown  (only proband) | het | WES |
| 23 | 00305907 | *SMN1* (NM_000344.3) | Exon 7 and 8 Del | P | Spinal muscular atrophy-1 [MIM:253300] | AR | Inherited (from father + mother) | hom | TES panel |
| 24 | 00305908 | *MMACHC* (NM_015506.3) | c.658_660delAAG;658_660delAAG | P | Methylmalonic aciduria and homocystinuria, cblC type [MIM:277400] | AR | Inherited (from father + mother) | hom | TES panel |
| 25 | 00305916 | *OTC* (NM_000531.6) | c.540G>C | LP | Ornithine transcarbamylase deficiency [MIM:311250] | XR | Inherited  (from mother) | het | TES panel |
| 26 | 00305917 | *CYP21A2* (NM_000500.9) | c.293-13C>G;  c.IVS2-12 C/A>G | LP  LP | Adrenal hyperplasia, congenital, due to 21-hydroxylase deficiency [MIM:201910] | AR | Unkown  (only proband) | het | TES panel |
| 27 | 00305929 | *MUT* (NM_000255.4) | c.1677-1G>A;  c.1106G>A | P  P | Methylmalonic aciduria, mut(0) type [MIM:251000] | AR | Inherited (from father + mother) | het | TES panel |
| 28 | 00305930 | *TSC2* (NM_000548.5) | c.1832G >A | LP | Tuberous sclerosis-2 [MIM:613254] | AD | Unkown  (only proband) | het | TES panel |

Table S1 Continued

| ID | LOVD  Individual ID | Gene(s) | Variants | Variant class (LP/P/VUS) | Disease(s) | Inheritance  pattern | Segregation | Zygosity | Molecular diagnostic technology |
| --- | --- | --- | --- | --- | --- | --- | --- | --- | --- |
| 29 | 00305931 | *ACADVL* (NM_000018.4) | c.228_231dup;  c.878+1G>C | LP  LP | VLCAD deficiency [MIM:201475] | AR | Inherited (from father + mother) | het | TES panel |
| 30 | 00305932 | *OTC* (NM_000531.6) | c.176T>C | LP | Ornithine transcarbamylase deficiency [MIM:311250] | XR | Inherited  (from mother) | hemi | TES panel |
| 31 | 00305933 | *KCNJ11* (NM_000525.3) | c.602G>A | P | Diabetes Mellitus, Permanent Neonatal [MIM:606176] | AD | De novo | het | TES panel |
| 32 | 00305934 | *EDA* (NM_001399.5) | c.730C>T | P | Ectodermal dysplasia 1, hypohidrotic, X-linked [MIM:305100] | XR | Inherited  (from mother) | hemi | WES |
| 33 | 00305935 | *IL10RA* (NM_001558.4) | c.301C>T;  c.1283delC | P  LP | Inflammatory bowel disease 28, early onset, autosomal recessive [MIM:613148] | AR | Inherited (from father + mother) | het | WES |
| 34 | 00305936 | *PEX26* (NM_017929.6) | c.29delC;  c.359T>G | LP  LP | Peroxisome biogenesis disorder 7A (Zellweger)[MIM:614872] | AR | Inherited (from father + mother) | het | TES panel |
| 35 | 00305937 | *MYO5B* (NM_001080467.3) | c.1306G>T;  c.3190C>T | LP  LP | Microvillus inclusion disease [MIM:251850] | AR | Inherited (from father + mother) | het | WES |
| 36 | 00305938 | *KMT2D* (NM_003482.3) | c.11429T>A | LP | Kabuki syndrome 1[MIM:147920] | AD | De novo | het | WES |
| 37 | 00305939 | *JAK3* (NM_000215.3) | c.3050T>C;  c.1744C>T | LP  P | Severe combined immunodeficiency [MIM:600802] | AR | Inherited (from father + mother) | het | WES |
| 38 | 00305940 | *IL2RG* (NM_000206.3) | c.421C>T | P | Combined immunodeficiency, X-linked, moderate ; X-linked severe combined immunodeficiency[MIM:312863] | XR | Inherited  (from mother) | hemi | WES |
| 39 | 00306845 | *PCSK1* (NM_000439.5) | c.1777G>A; 1777G>A | LP | Obesity with impaired prohormone processing[MIM:600955] | AR | Inherited (from father + mother) | hom | WES |
| 40 | 00305941 | *GTPBP3* (NM_001195422.1) | c.253C>T;  c.479C>T | P  LP | Combined oxidative phosphorylation deficiency 23[MIM:616198] | AR | Inherited (from father + mother) | het | WES |
| 41 | 00305942 | *ALOX12B* (NM_001139.3) | c.1405C>T  c.163_173delGGGCACTGCAC; | VUS  LP | Ichthyosis, congenital, autosomal recessive 2[MIM:242100] | AR | Inherited  (from father) | het | TES panel |
| 42 | 00305943 | *GSS* (NM_000178.4) | c.738dupG; a repetitive sequence in exon 3 | P | Glutathione synthetase deficiency[MIM:266130] | AR | Inherited (from father + mother) | het | TES panel |

Table S1 Continued

| ID | LOVD  Individual ID | Gene(s) | Variants | Variant class (LP/P/VUS) | Disease(s) | Inheritance  pattern | Segregation | | Zygosity | Molecular diagnostic technology |
| --- | --- | --- | --- | --- | --- | --- | --- | --- | --- | --- |
| 43 | 00305944 | *EPCAM* (NM_002354.3) | c.96C>A;  c.823delG | LP  LP | Diarrhea 5, with tufting enteropathy, congenital[MIM:613217] | AR | Inherited (from father + mother) | het | | WES |
| 44 | 00305945 | *AQP2* (NM_000486.5) | c.454C> T;  c.202A>T | LP  LP | Diabetes insipidus, nephrogenic [MIM:125800] | AR | Inherited (from father + mother) | het | | WES |
| 45 | 00305946 | *F13A1* (NM_000129.4) | c.2015G>A;  c.1352_1353del | LP  LP | Factor XIIIA deficiency [MIM:613225] | AR | Unkown  (only proband) | het | | TES panel |
| 46 | 00305947 | *MTM1* (NM_000252.3) | c.614C>T | P | Myotubular myopathy, X-linked [MIM:310400] | XR | Inherited  (from mother) | hemi | | WES |
| 47 | 00305948 | *ABCA3* (NM_001089.3) | c.3799A>G;  c.127C>T | LP  P | Surfactant metabolism dysfunction, pulmonary, 3[MIM:610921] | AR | Inherited (from father + mother) | het | | TES panel |
| 48 | 00306834 | Xp11.23-p11.22 dup | chrXp11.23-p11.22 dup | LP | Chromosome Xp11.23-p11.22 duplication syndrome[MIM:300801] | XD | Unkown  (only proband) | het | | WES |
| 49 | 00305949 | *SCNN1G* (NM_001039.4) | c.116A>G;  c.1415C>T | LP  LP | Pseudohypoaldosteronism, type I  [MIM:264350] | AR | Inherited  (from father)/  De novo | het | | TES panel |
| 50 | 00305950 | *JAG1* (NM_000214.3) | c.2347delA | LP | Alagille syndrome 1[MIM:118450] | AD | De novo | het | | TES panel |
| 51 | 00306835 | 11q24.1-q25 del | Chr 11q24.1-q25 del | P | Jacobsen syndrome[MIM:147791] | Isolated cases | De novo | het | | WES |
| 52 | 00306819 | 16p11.2-p12.2 del | Chr 16p11.2-p12.2 del | P | Chromosome 16p12.2-p11.2 deletion syndrome [MIM:613604] | Isolated cases | De novo | het | | WES |
| 53 | 00305951 | *DMD* (NM_004006.2) | c.7391_7394del | LP | Duchenne muscular dystrophies [MIM:310200] | XL | De novo | hemi | | WES |
| 54 | 00305952 | *IL10RA* (NM_001558.4) | c.301C>T;  c.537G>A | P  P | Inflammatory bowel disease 28, early onset, autosomal recessive [MIM:613148] | AR | Inherited (from father + mother) | het | | WES |
| 55 | 00306120 | *STAR* (NM_000349.3) | c.772C>T;  c.556A>G | LP  LP | Lipoid adrenal hyperplasia [MIM:201710] | AR | Unkown  (only proband) | het | | WES |
| 56 | 00306121 | *IKBKG*( NM_003639.4) | c.1110delinsTT | LP | Immunodeficiency 33[MIM:300636] | XR | Inherited  (from mother) | hemi | | WES |

Table S1 Continued

| ID | LOVD  Individual ID | Gene(s) | Variants | Variant class (LP/P/VUS) | Disease(s) | Inheritance  pattern | Segregation | Zygosity | Molecular diagnostic technology |
| --- | --- | --- | --- | --- | --- | --- | --- | --- | --- |
| 57 | 00306123 | *G6PD* (NM_000402.3) | c.185A>G | P | Hemolytic anemia, G6PD deficient (favism)[MIM:300908] | XD | Inherited  (from mother) | hemi | WES |
| 58 | 00306125 | *JAG1* (NM_000214.3) | c.532delC | P | Alagille syndrome 1[MIM:118450] | AD | De novo | het | WES |
| 59 | 00306126 | *ACADVL* (NM_000018.3) | c.1280G>A;  c.1505T>A | LP  LP | VLCAD deficiency [MIM:201475] | AR | Unkown  (only proband) | het | TES panel |
| 60 | 00306128 | *CHRNA1* (NM_000079.3) | c.1063G>T | LP | Myasthenic syndrome, congenital, 1A, slow-channel [MIM:601462] | AD | Unkown  (only proband) | het | WES |
| 61 | 00306836 | *PRKAR1A* (NM_01276290.1) | Exon 10 del | VUS | Acrodysostosis 1, with or without hormone resistance[MIM:101800] | AD | Inherited  (from father) | het | WES |
| 62 | 00306129 | *KCNJ11* (NM_000525.3) | c.527G>T;  c.426C>G | LP  LP | Hyperinsulinemic hypoglycemia, familial, 2[MIM:601820] | AR | Inherited (from father + mother) | het | WES |
| 63 | 00306130 | *HRAS* (NM_005343.4) | c.37G>T | P | Costello syndrome[MIM:16835863] | AD | De novo | het | WES |
| 64 | 00306131 | *COL7A1* (NM_000094.3) | c.3625_3635del; 3625_3635del | P | Epidermolysis bullosa dystrophica, AR [MIM:226600] | AR | Inherited (from father + mother) | hom | WES |
| 65 | 00306837 | *IKBKG* ( NM_003639.4) | Exon 4-10 del | P | Incontinentia pigmenti[MIM:308300] | XD | Inherited  (from mother) | het | WES |
| 66 | 00306132 | *FGFR3* (NM_000142.4) | c. 1138G>A | P | Hypochondroplasia[MIM:146000] | AD | De novo | het | WES |
| 67 | 00306133 | *SCN8A* (NM_014191.4) | c. 1391A>G | LP | Epileptic encephalopathy, early infantile, 13 [MIM:614558] | AD | Unkown  (only proband) | het | WES |
| 68 | 00306134 | *SHOC2* (NM_007373.4) | c.4A>G | P | Noonan-like syndrome with loose anagen hair[MIM:607721] | AD | De novo | het | WES |
| 69 | 00306838 | *SMN1* (NM_000344.3) | Exon 7-8 del | P | Spinal muscular atrophy-1[MIM:253300] | AR | Inherited (from father + mother) | hom | WES |
| 70 | 00306135 | *CHRNA2* (NM_000742.4) | c. 1073G>T | VUS | Epilepsy, nocturnal frontal lobe, 4 [MIM:610353] | AD | Inherited  (from mother) | het | WES |
| 71 | 00306136 | *SCNN1A* (NM_001038.6) | c.1439+1G>C;  c.875+1G>A | LP  LP | Pseudohypoaldosteronism, type I [MIM:264350] | AR | Inherited (from father + mother) | het | WES |
| 72 | 00306137 | *SCNN1A* (NM_001038.6) | c.1439+1G>C;  c.875+2G>A | LP  LP | Pseudohypoaldosteronism, type I [MIM:264350] | AR | Inherited (from father + mother) | het | WES |

Table S1 Continued

| ID | LOVD  Individual ID | Gene(s) | Variants | Variant class (LP/P/VUS) | Disease(s) | Inheritance  pattern | Segregation | Zygosity | Molecular diagnostic technology |
| --- | --- | --- | --- | --- | --- | --- | --- | --- | --- |
| 73 | 00306830 | 15q11.2-13.1 del | Chr15q11.2-13.1 del | P | Prader-Willi syndrome due to paternal deletion of 15q11q13 type 2 [MIM:176270] | Imprinted disease | Inherited  (from father) | het | WES |
| 74 | 00306831 | 15q11.2-13.1 del | Chr15q11.2-13.1 del | P | Prader-Willi syndrome due to paternal deletion of 15q11q13 type 2 [MIM:176270] | Imprinted disease | Inherited  (from father) | het | WES |
| 75 | 00306138 | *DNAH5* (NM_001369.2) | c.11029G>A;  c.10442G>A | LP  LP | Ciliary dyskinesia, primary, 3, with or without situs inversus [MIM:608644] | AR | Inherited (from father + mother) | het | WES |
| 76 | 00306139 | *KRT14* (NM_000526.5) | c.374G>A | LP | Epidermolysis bullosa simplex, Dowling-Meara type[MIM:131760] | AD | Unkown  (only proband) | het | WES |
| 77 | 00306140 | *PEX1* (NM_000466.3) | c.2050C>T;  c.782_783 del | P  P | Peroxisome biogenesis disorder 1A (Zellweger)[MIM:214100] | AR | Inherited (from father + mother) | het | WES |
| 78 | 00306142 | *EZH2* (NM_004456.5) | c.2050C>T | P | Weaver syndrome[MIM:277590] | AD | De novo | het | WES |
| 79 | 00306143 | *ZC4H2* (NM_018684.4) | c.352C>T | LP | Wieacker-Wolff syndrome [MIM:314580] | XR | De novo | het | WES |
| 80 | 00306144 | *IGHMBP2* (NM_002180.3) | c.2356del;  c.344C>T | LP  LP | Neuronopathy, distal hereditary motor, type VI [MIM:604320]; Charcot-Marie-Tooth disease, axonal, type 2S[MIM:616155] | AR | Unkown  (only proband) | het | WES |
| 81 | 00306183 | *ABCC8* (NM_000352.6) | c.2553_2555del;  c.824G>A | P  P | Hyperinsulinemic hypoglycemia, familial, 1[MIM:256450] | AR | Inherited (from father + mother) | het | WES |
| 82 | 00306184 | *COL7A1* (NM_000094.3) | c.6181-2A>G | VUS | EBD, Bart type[MIM:132000] | AD | Inherited  (from father) | het | WES |
| 83 | 00306185 | *MYH7* (NM_000257.4) | c.5655G>A | VUS | Cardiomyopathy, dilated, 1S,Left ventricular noncompaction 5 [MIM:613426]; Cardiomyopathy, hypertrophic, 1 [MIM:192600];  Laing distal myopathy [MIM:160500]; Myopathy, myosin storage, autosomal dominant[MIM:608358];  Scapuloperoneal syndrome, myopathic type [MIM:181430] | AD | Inherited  (from father) | het | WES |
| 84 | 00306186 | *SPINK5* (NM_006846.4) | c.377_378del;  c.2468dup | P  P | Netherton syndrome[MIM:256500] | AR | Inherited (from father + mother) | het | WES |

Table S1 Continued

| ID | LOVD  Individual ID | Gene(s) | Variants | Variant class (LP/P/VUS) | Disease(s) | Inheritance  pattern | Segregation | Zygosity | Molecular diagnostic technology |
| --- | --- | --- | --- | --- | --- | --- | --- | --- | --- |
| 85 | 00306187 | *PACS2* (NM_001100913.3) | c.625G>A | P | Epileptic encephalopathy, early infantile, 66 [MIM:618067] | AD | De novo | het | WES |
| 86 | 00306188 | *COL7A1* (NM_000094.3) | c.5980-2A>G | P | Epidermolysis bullosa dystrophica, AD [MIM:131750] | AD | De novo | het | WES |
| 87 | 00306189 | *FBN2* (NM_001999.4) | c.3725-15A>G | P | Contractural arachnodactyly, congenital [MIM:121050] | AD | De novo | het | WES |
| 88 | 00306190 | *PLOD1* (NM_000302.4) | c.1462del;  c.2T>C | P  LP | Ehlers-Danlos syndrome, kyphoscoliotic type, 1[MIM:225450] | AR | Inherited (from father + mother) | het | WES |
| 89 | 00306191 | *COL7A1* (NM_000094.3) | c.6929G>A | VUS | Epidermolysis bullosa dystrophica, AD [MIM:131750] | AD | Inherited  (from mother) | het | WES |
| 90 | 00306192 | *IKBKG* ( NM_001099856.5) | Exion 4-10 del | P | Incontinentia pigmenti[MIM:308300] | XD | Inherited  (from mother) | het | WES |
| 91 | 00306193 | *CREBBP* (NM_004380.3) | c.4990C>T | LP | Rubinstein-Taybi syndrome 1 [MIM:180849] | AD | De novo | het | WES |
| 92 | 00306194 | *MMACHC* (NM_015506.3) | c.80A>G;  c.658_660del | LP  P | Methylmalonic aciduria and homocystinuria cblC type [MIM:277400] | AR | Inherited (from father + mother) | het | WES |
| 93 | 00306195 | *RPL5* (NM_000969.5) | c.95C>T | LP | Diamond-Blackfan anemia 6 [MIM:612561] | AD | De novo | het | WES |
| 94 | 00306196 | *ABCC8* (NM_000352.6) | c.2475+1G>A;  c.4460_4463dup | P  P | Hyperinsulinemic hypoglycemia, familial, 1[MIM:256450] | AR | Inherited (from father + mother) | het | WES |
| 95 | 00306197 | *OTC* (NM_000531.6) | c.803T>C | P | Ornithine transcarbamylase deficiency [MIM:311250] | XR | Inherited  (from mother) | hemi | WES |
| 96 | 00306198 | *KAT6B* (NM_012330.4) | p.5027del | P | SBBYSS syndrome[MIM:603736] | AD | De novo | het | WES |
| 97 | 00306199 | *SMN1* (NM_000344.3) | Exon 7and 8 del | P | Spinal muscular atrophy-1 [MIM:253300] | AR | Inherited (from father + mother) | hom | WES |
| 98 | 00306200 | *DUOX2* (NM_014080.4) | c.3693+1G>T;  c.2654G>A | P  P | Thyroid dyshormonogenesis 6 [MIM:607200] | AR | Inherited (from father + mother) | het | WES |
| 99 | 00306201 | *CHD7* (NM_017780.4) | c.6292C>T | P | CHARGE syndrome[MIM:2148000] | AD | De novo | het | WES |

Table S1 Continued

| ID | LOVD  Individual ID | Gene(s) | Variants | Variant class (LP/P/VUS) | Disease(s) | Inheritance  pattern | Segregation | Zygosity | Molecular diagnostic technology |
| --- | --- | --- | --- | --- | --- | --- | --- | --- | --- |
| 100 | 00306202 | *CPS1* (NM_001875.5) | c.3784C>T;  c.3734T>A | P  LP | Carbamoylphosphate synthetase I deficiency[MIM:237300] | AR | Inherited (from father + mother) | het | WES |
| 101 | 00306203 | *AMER1* (NM_152424.4） | c.2082C>A | LP | Osteopathia striata with cranial sclerosis [MIM:300373] | XD | Inherited  (from mother) | het | WES |
| 102 | 00306204 | *DNAH11* (NM_001277115.2) | c.12937dup;  c.6143C>G | P  LP | Ciliary dyskinesia, primary, 7, with or without situs inversus[MIM:611884] | AR | Inherited (from father + mother) | het | WES |
| 103 | 00306205 | *MYH7* (NM_000257.3) | c.5704G>C | VUS | Laing distal myopathy [MIM:160500] | AD | Inherited  (from father) | het | WES |
| 104 | 00306206 | *CYP21A2* (NM_000500.7） | CYP21A2, 30kb del | LP | Adrenal hyperplasia, congenital, due to 21-hydroxylase deficiency[MIM:201910] | AR | Unkown  (only proband) | hom | WES |
| 105 | 00306207 | *CXCR4* (NM_003467.2) | c.685T>A | VUS | WHIM syndrome [MIM:193670] | AD | Inherited  (from mother) | het | WES |
| 106 | 00306208 | 15q11.2-13.1 del | Chr15q11.2-13.1 del | P | Prader-Willi syndrome due to paternal deletion of 15q11q13 type 2 [MIM:176270] | Imprinted disease | Inherited  (from father) | het | WES |
| 107 | 00306209 | *RPS19* (NM_001022.3) | c.3G>A | P | Diamond-Blackfan anemia 1 [MIM:105650] | AD | De novo | het | WES |
| 108 | 00306839 | Chromosome 18 duplication | Chromosome 18 duplication | LP | Trisomy 18-liked syndrome[MIM:601161] | Isolated cases | Unkown  (only proband) | het | WES |
| 109 | 00306210 | *NPHS1* (NM_004646.3) | c.3027C>G; 3027C>G | P | Nephrotic syndrome, type 1[MIM:256300] | AR | Inherited (from father + mother) | hom | WES |
| 110 | 00306832 | 15q11.2-13.1 del | Chr 15q11.2-13.1 del | P | Prader-Willi syndrome due to paternal deletion of 15q11q13 type 2 [MIM:176270] | Imprinted disease | Inherited  (from father) | het | WES |
| 111 | 00306211 | *COL17A1* (NM_000494.3) | c.2363dupG;  c.3301C>T | LP  LP | Epidermolysis bullosa, junctional, localisata variant [MIM:226650] | AR | Unkown  (only proband) | het | WES |
| 112 | 00306212 | *ALG12* (NM_024105.3) | c.432C>A；  c.904T>C | P  LP | Congenital disorder of glycosylation, type Ig [MIM:607143] | AR | Inherited (from father + mother) | het | WES |
| 113 | 00306213 | *KMT2D* (NM_003482.3) | c.14382G>A | LP | Kabuki syndrome 1[MIM:147920] | AD | De novo | het | WES |
| 114 | 00306840 | 20q13.33 del | Chr 20q13.33 del | LP | Epilepsy, nocturnal frontal lobe, 1 [MIM:600513] | AD | Unkown  (only proband) | het | WES |

Table S1 Continued

| ID | LOVD  Individual ID | Gene(s) | Variants | Variant class (LP/P/VUS) | Disease(s) | Inheritance  pattern | Segregation | Zygosity | Molecular diagnostic technology |
| --- | --- | --- | --- | --- | --- | --- | --- | --- | --- |
| 115 | 00306214 | *EFTUD2* (NM_004247.3) | c.869+1G>A | LP | Mandibulofacial dysostosis, Guion-Almeida type [MIM:610536] | AD | Unkown  (only proband) | het | WES |
| 116 | 00306215 | *GLDC* (NM_000170.2) | c.862-2A>G;  c.2098C>G | P  P | Glycine encephalopathy[MIM:605899] | AR | Inherited (from father + mother) | het | WES |
| 117 | 00306216 | *CYP21A2* (NM_000500.7) | c.293-13A>G;  c.955C>T | P  P | Adrenal hyperplasia, congenital, due to 21-hydroxylase deficiency [MIM:201910] | AR | De novo  Inherited(mother) | het | WES |
| 118 | 00311380 | Distal duplication 16q | Distal duplication 16q | LP | Distal trisomy 16q | Isolated cases | Unkown  (only proband) | het | WES |
| 119 | 00306217 | *KCNJ11* (NM_000525.3) | c.1006delA;1006delA | P | Hyperinsulinemic hypoglycemia, familial, 2 [MIM:601820] | AR | Inherited (from father + mother) | hom | WES |
| 120 | 00306218 | *MYO5B* (NM_001080467.2) | c.2T>C;  c.1201C>G | VUS  VUS | Microvillus inclusion disease [MIM:251850] | AR | Unkown  (only proband) | het | WES |
| 121 | 00311381 | 12p13.33-11.21 dup | Chr 12p13.33-11.21 dup | LP | Trisomy 12p | Isolated cases | Unkown  (only proband) | het | WES |
| 122 | 00306219 | *GP1BA* (NM_000173.6) | c.737G>T | LP | Bernard-Soulier syndrome, type A2 (dominant)[MIM:153670] | AD | Inherited  (from father) | het | WES |
| 123 | 00306220 | *RASA1* (NM_002890.2) | c.2828T>C | LP | Capillary malformation-arteriovenous malformation 1 [MIM:608354] | AD | Inherited  (from father) | het | WES |
| 124 | 00311382 | 21q22.12-22.13 del | Chr 21q22.12-22.13 del | LP | Monosomy 21 | Isolated cases | Unkown  (only proband) | het | WES |
| 125 | 00306221 | *RAF1* (NM_002880.3) | c.770C>T | P | Noonan syndrome 5 [MIM:611553] | AD | De novo | het | WES |
| 126 | 00306841 | 15q11.2-13.1 del | Chr 15q11.2-13.1 del | P | Prader-Willi syndrome due to paternal deletion of 15q11q13 type 2 [MIM:176270] | Imprinted disease | Inherited  (from father) | het | WES |
| 127 | 00306222 | *SCN2A* (NM_021007.2) | c.2305A>G | LP | Seizures, benign familial infantile, 3 [MIM:607745] | AD | De novo | het | WES |
| 128 | 00306223 | *TGFBR2* (NM_003242.5) | c.95-2A>G | LP | Loeys-Dietz syndrome 2[MIM:610168] | AD | Inherited  (from mother) | het | WES |
| 129 | 00306842 | 11q24.1-q25 del | Chr 11q24.1-q25 del | LP | Jacobsen syndrome [MIM:147791] | Isolated cases | Unkown  (only proband) | het | WES |

Table S1 Continued

| ID | LOVD  Individual ID | Gene(s) | Variants | Variant class (LP/P/VUS) | Disease(s) | Inheritance  pattern | Segregation | Zygosity | Molecular diagnostic technology |
| --- | --- | --- | --- | --- | --- | --- | --- | --- | --- |
| 130 | 00306224 | *KCNQ2* (NM_172107.2) | c.2513_2514delAG | P | Seizures, benign neonatal, 1 [MIM:121200] | AD | De novo | het | WES |
| 131 | 00306843 | 2q37.3 del | Chr 2q37.3 del | LP | 2q37 microdeletion syndrome [MIM:600430] | AD | Unkown  (only proband) | het | WES |
| 132 | 00306225 | *NPC1* (NM_000271.4) | c.3206T>A;  c.3467A>G | VUS  VUS | Niemann-Pick disease, type C1[MIM:257220];  Niemann-Pick disease, type D [MIM:257220] | AR | Unkown  (only proband) | het | WES |
| 133 | 00306226 | *EYA1* (NM_000503.5) | c.229C>T | LP | Anterior segment anomalies with or without cataract,Branchiootic syndrome 1 [MIM:602588];  Branchiootorenal syndrome 1, with or without cataracts[MIM:113650];  Otofaciocervical syndrome [MIM:166780] | AD | Inherited  (from mother) | het | WES |
| 134 | 00306227 | *PPP2R5D* (NM_006245.3) | c.598G>A | P | Mental retardation, autosomal dominant 35 [MIM:616355] | AD | De novo | het | WES |
| 135 | 00306228 | *NIPBL* (NM_133433.3) | c.6892C>T | LP | Cornelia de Lange syndrome 1 [MIM:122470] | AD | Unkown  (only proband) | het | WES |
| 136 | 00306229 | *PTPN11* (NM_002834.3) | c.1517A>C | P | Noonan syndrome 1 [MIM:163950];  LEOPARD syndrome 1[MIM:151100]; | AD | De novo | het | WES |
| 137 | 00306230 | *PRF1* (NM_001083116.1) | c.65delC；  c.1298C>T | P  LP | Hemophagocytic lymphohistiocytosis, familial, 2 [MIM:603553] | AR | Inherited (from father + mother) | het | WES |
| 138 | 00306844 | Xp11.23-p11.22 dup | chrXp11.23-p11.22 dup | LP | Chromosome Xp11.23-p11.22 duplication syndrome[MIM:300801] | XD | Unkown  (only proband) | het | WES |
| 139 | 00306231 | *KMT2D* (NM_003482.3) | c.6595delT | LP | Kabuki syndrome 1[MIM:147920] | AD | Unkown  (only proband) | het | WES |
| 140 | 00306232 | *DDX58* (NM_014314.3) | c.738C>A | VUS | Singleton-Merten syndrome 2 [MIM:616298] | AD | Inherited  (from father) | het | WES |
| 141 | 00306233 | *KMT2D* (NM_003482.3) | c.5531delC | P | Kabuki syndrome 1[MIM:147920] | AD | De novo | het | WES |
| 142 | 00306234 | *MYBPC3* (NM_000256.3) | c.1153_1168del | LP | Left ventricular noncompaction 10 [ MIM:615396];  Cardiomyopathy, hypertrophic, 4 [MIM:115197] | AD | Inherited  (from mother) | het | WES |

Abbreviations: P, pathogenic varivants; LP, likely pathogenic variants; VUS, variants of unkown clinical significance; MIM, Mendelian Inheritance in Man; AD, autosomal dominant inheritance

disease; AR, autosomal recessive inheritance disease; XD, X-linked dominant inheritance disease; XR, X-linked recessive inheritance disease; het, heterozygous; hemi, hemizygous; hom, homozygous; WES, whole-exome sequencing, MLAP, multiplex ligation-dependent probe amplification, CMA,chromosomal microarray analysis,CNV, copy number variation.

Mutations that have been reported their pathogenicity previously were referred to “P” (pathogenic varivants);

All variants, including rearrangements,stop codon-introducing (nonsense) and insertion/deletion (indel) were regarded as null alleles, abolishing production of the corresponding protein from

the affected allele.

SIFT and PolyPhen-2 are two pathogenicity predictions used to evaluate putative pathogenicity of novel non-synonymous coding variants (unreported previously);Human Splicing Finder is used to predict transcript splicing site of splice mutations(unreported previously) and evaluate putative pathogenicity.

**Table S2 Characteristics of patients with a positive diagnosis molecular and without the molecular diagnosis**

| Clinical Factors | Infants without a positive molecular diagnosis(n=165) | Infants with a positive molecular diagnosis(n=142) | Statistical value | P Value |
| --- | --- | --- | --- | --- |
| Male [n (%)] | 95(57.58) | 88(61.97) | *χ^2^*=0.61 | 0.434 |
| Age of enrollment [*M*(*P*_25_~*P*_75_)] | 6(1-24.5) | 9.5(1-27.25) | Z=-0.89 | 0.375 |
| Gestational age ($\bar{x}\pm s$) | 36.89±3.47 | 38.16±2.41 | t=-3.78 | <0.001^a^ |
| GA < 37weeks [n (%)] | 58(35.15) | 32(22.54) | *χ^2^*=5.86 | 0.015^b^ |
| Birth weight ($\bar{x}\pm s$) | 2703±810.05 | 3118.14±738.21 | t=-4.66 | <0.001^a^ |
| Prenatal anomalies [n (%)] | 17(10.30) | 12(8.45) | *χ^2^*=0.31 | 0.58 |
| Family history [n (%)] | 7(4.24) | 7(4.93) | *χ^2^*=0.08 | 0.774 |
| Age of testing [*M*(*P*_25_~*P*_75_)] | 21(8-45) | 18.5(7-34.25) | Z=-1.23 | 0.218 |
| Length of stay [*M*(*P*_25_~*P*_75_)] | 14(8.5-34) | 13(7-25.25) | Z=-1.95 | 0.051 |
| TAT [*M*(*P*_25_~*P*_75_)] | 64(38-66) | 45.5(34-64) | Z=-3.20 | 0.001^c^ |
| 180d death [n (%)] | 31(18.79) | 50(35.21) | *χ^2^*=10.60 | 0.001^b^ |

^a^:P-value was obtained from t-test;

^b^:P-value was obtained from Pearson’s Chi-square test;

^c^: P-value was obtained from the Mann–Whitney U test.

Abbreviations: GA, Gestational age; TAT, turnaround time.

**Table S3 Characteristics of patients in different inclusion scenarios according to each variable**

| Clinical Factors | CH patients (n=95) | XH/SCMC patients (n=212) | Statistical value | P Value |
| --- | --- | --- | --- | --- |
| Male [n (%)] | 52(54.74) | 131(61.79) | *χ^2^*=1.356 | 0.244^b^ |
| Age of admission [*M*(*P*_25_~*P*_75_)],days | 2(0.1-16) | 13(1-30.25) | Z=-3.641 | <0.001^c^ |
| Gestational age [*M*(*P*_25_~*P*_75_)],weeks | 36.72±3.62 | 37.82±2.76 | Z=-2.345 | 0.019^c^ |
| GA < 37weeks [n (%)] | 37(38.95) | 54(25.47) | *χ^2^*=5.712 | 0.017^b^ |
| Birth weight ($\bar{x}\pm s$),g | 2795.21±916.47 | 2939.75±745.55 | t= -1.459 | 0.145^a^ |
| Prenatal anomalies [n (%)] | 1(1.05) | 28(13.21) | *χ^2^*=9.954 | 0.002^b^ |
| Family history [n (%)] | 0(0.00) | 14(6.60) | *χ^2^*(/) | / |
| Age of testing [*M*(*P*_25_~*P*_75_)],days | 12(4-25) | 23(10-45.75) | Z=-4.669 | <0.001^c^ |
| Length of stay [*M*(*P*_25_~*P*_75_)],days | 14(7-33) | 14(8-28) | Z=-0.092 | 0.927^c^ |
| TAT [*M*(*P*_25_~*P*_75_)],days | 48(35-78) | 55(35.25-66) | Z=-0.104 | 0.917^c^ |
| Age of confirmed diagnosis [M (P25~P75)], days | 62(42-97) | 81.5(66.25-100.75) | Z=-3.588 | <0.001^c^ |
| 180d death [n (%)],days | 28 | 53 | *χ^2^*=11.26 | 0.411^b^ |
| A positive molecular diagnosis [n(%)] | 42 | 100 | *χ^2^*=11.26 | 0.631^b^ |

^a^:P-value was obtained from t-test;

^b^:P-value was obtained from Pearson’s Chi-square test;

^c^: P-value was obtained from the Mann–Whitney U test.

Abbreviations: GA, Gestational age; TAT, turnaround time; CH: Children's Hospital, Shanghai Jiao Tong University School of Medicine; SCMC: Shanghai Children’s Medical Center, Shanghai Jiao Tong University School of Medicine.

**Table S4. Definitions of phenotype or mixed phenotypes related to a molecular diagnosis**

| HPO term | HPO ID | Clinical meaning |
| --- | --- | --- |
| Abnormality of metabolism/homeostasis | HP:0001939 |  |
| Hyperinsulinemic hypoglycemia | HP:0000825 | An increased concentration of insulin (>14.4 pmol/L [2 μU/mL]) & a decreased concentration of glucose in the blood (<2.7 mmol/L [50 mg/dL]) & oral feeding do not maintain normal glucose levels & hydrocortisone iv do not result in stable glucose levels. |
| Hyperkalemia AND Hyponatremia | HP:0002153 AND HP:0002902 | An abnormally increased potassium concentration (>5.5mmol/L) in the blood and an abnormally decreased sodium concentration (<135mmol/L) in the blood. |
| Elevated serum creatine phosphokinase | HP:0003236 | Serum creatine kinase is 100-200 times the normal level. |
| Metabolic acidosis OR Lactic acidosis | HP:0001942 OR  HP:0003128 | Acid accumulation or depletion of base in the body due to buildup of metabolic acids.  OR An abnormal buildup of lactic acid in the body, leading to acidification of the blood and other bodily fluids. |
| Hyperammonemia | HP:0001987 | An increased concentration of ammonia (>200umol/L) in the blood. |
| Abnormality of the nervous system  Seizures    Neonatal hypotonia  Abnormality of the respiratory system  Respiratory failure  Abnormality of the cardiovascular system  Cardiomyopathy  Arrhythmia  Abnormality of the integument  Blister  Ichthyosis  Abnormality of blood and blood-forming tissues  Thrombocytopenia  Anemia requiring red cell transfusion  Congenital anomalies (CA)  Newborns with single-system CA  Newborns with multiple CA  Abnormality of the immune system  recurrent infections  protracted diarrhea  Leukocytosis  Leukopenia  Abnormal immunoglobulin level  Cholestasis | HP:0000707  HP:0001250  HP: 00013191  HP:0002086  HP:0002878  HP:0001626  HP:0001638  HP:0011675  HP:0001574  HP:0008064  HP:0008064  HP:0001871  HP: 0001873  HP: 0001903  HP:0000152 OR HP:0000598 OR  HP:0000924 OR  HP:0003011 OR  HP:0040064 OR  HP:0011603 OR HP:0004307  HP:0002715  HP:0002719  HP:0004385  HP:0001974  HP:0001882  HP:0010701 | An intermittent `abnormality of the central nervous system` due to a sudden, excessive, disorderly discharge of cerebral neurons and characterized clinically by some combination of disturbance of sensation, loss of consciousness, impairment of psychic function, or convulsive movements.  Muscular hypotonia (abnormally low muscle tone) manifesting in the neonatal period.  A severe form of respiratory insufficiency characterized by inadequate gas exchange such that the levels of oxygen or carbon dioxide cannot be maintained within normal limits. (not related to infection)  A myocardial disorder in which the heart muscle is structurally and functionally abnormal, in the absence of coronary artery disease, hypertension, valvular disease and congenital heart disease sufficient to cause the observed myocardial abnormality.  Only ventricular arrhythmia (HP:0004308) and supraventricular arrhythmia (HP:0005115) include.  Onset birth-newborn period abnormal blistering of the skin.  Onset birth-newborn period ichthyosis.  A reduction in the number of circulating `thrombocytes`.  Anemia sufficiently severe as to require red cell transfusion (WHO Grade 3 or 4).  Abnormalities affecting a single organ, organ system or body part.  Abnormalities affecting more than one organ system or body part.  Increased susceptibility to infections.  Severe, progressive infantile onset inflammatory bowel disease  An abnormal increase in the number of leukocytes in the blood.  An abnormal decreased number of leukocytes in the blood.  An abnormal deviation from normal levels of immunoglobulins in blood.  Impairment of bile flow due to obstruction in bile ducts. |

Note: This description, based on cases published in biomedical literatures and our cohort, uses the phenotypic abnormalities referenced in the Human Phenotype Ontology (HPO). Seven root HPO categories ‘Phenotypic abnormality’ and two separate HPO categories are listed. Listed under each top-branch header are the unique HPO-based abnormalities that were reported among the cohort of 307 infants in our study.

Abbreviations: HPO, human phenotype ontology; HP, human phenotype.

**Table S5. The Frequency Distribution of 7 top-level branching of HPO terms and 5 separate HPO terms in 307 subjects**

| HPO term | Molecular diagnosis | No molecular diagnosis | Total |
| --- | --- | --- | --- |
| Abnormality of metabolism/homeostasis |  |  |  |
| Individuals with the term | 46 | 20 | 66 |
| Individuals without the term | 96 | 145 | 241 |
| Abnormality of the nervous system |  |  |  |
| Individuals with the term | 40 | 11 | 51 |
| Individuals without the term | 102 | 154 | 256 |
| Abnormality of the respiratory system |  |  |  |
| Individuals with the term | 12 | 12 | 24 |
| Individuals without the term | 130 | 153 | 283 |
| Congenital anomalies |  |  |  |
| Individuals with the term | 24 | 47 | 71 |
| Individuals without the term | 118 | 118 | 236 |
| Neonatal hypotonia |  |  |  |
| Individuals with the term | 36 | 10 | 46 |
| Individuals without the term | 106 | 155 | 261 |
| Abnormality of the integument |  |  |  |
| Individuals with the term | 15 | 1 | 16 |
| Individuals without the term | 127 | 164 | 291 |
| Abnormality of blood and blood-forming tissues |  |  |  |
| Individuals with the term | 12 | 5 | 17 |
| Individuals without the term | 130 | 160 | 290 |
| Abnormality of the immune system |  |  |  |
| Individuals with the term | 38 | 1 | 39 |
| Individuals without the term | 104 | 164 | 268 |
| Cholestasis |  |  |  |
| Individuals with the term | 3 | 2 | 5 |
| Individuals without the term | 139 | 163 | 302 |
| Abnormality of the cardiovascular system |  |  |  |
| Individuals with the term | 2 | 6 | 8 |
| Individuals without the term | 140 | 159 | 299 |
| Metabolism/homeostasis & Nervous system |  |  |  |
| Individuals with the term | 10 | 2 | 12 |
| Individuals without the term | 132 | 163 | 295 |
| Nervous system & Congenital anomalies |  |  |  |
| Individuals with the term | 7 | 3 | 10 |
| Individuals with the term | 135 | 162 | 297 |

Note: Seven top-level branching of HPO terms include abnormality of metabolism/homeostasis, abnormality of the nervous system, abnormality of the respiratory system, abnormality of the integument, abnormality of blood and blood-forming tissues, abnormality of the immune system and abnormality of the cardiovascular system. Three separate HPO terms include congenital anomalies, neonatal hypotonia and cholestasis.

Abbreviations: HPO, human phenotype ontology; HP, human phenotype.

**Table S6. Multivariable logistic regression analysis for a positive diagnosis.**

| Independent predictors | Odds Ratio (95% CI) | *P* value |
| --- | --- | --- |
| Abnormality of metabolism/homeostasis | 3.429 (1.678-7.008) | 0.001 |
| Neonatal hypotonia | 3.523 (1.418-8.753) | 0.007 |
| Abnormality of the integument | 37.346 (4.566-305.488) | 0.001 |
| Abnormality of the immune system | 29.632 (3.697-237.512) | 0.001 |
| the number of phenotypes | 1.925 (1.355-2.735) | <0.001 |

**Table S7. The area under curve (AUC) and Youden’s index of five independent variables.**

| Independent predictors | AUC (95% CI) | *P* value | Youden’s index |
| --- | --- | --- | --- |
| Abnormality of metabolism/homeostasis | 0.395 (0.331-0.459) | 0.002 | having poor discriminatory value |
| Neonatal hypotonia | 0.404 (0.339-0.468) | 0.004 | having poor discriminatory value |
| Abnormality of the integument | 0.450 (0.385-0.515) | 0.133 | - |
| Abnormality of the immune system | 0.369 (0.306-0.433) | <0.001 | having poor discriminatory value |
| the number of phenotypes | 0.777 (0.726-0.829) | <0.001 | minimally accurate |

The data are presented as point estimation with 95% confidence interval.

We defined areas under the ROC curves (AUCs) < 0.7 as having poor discriminatory value, 0.7-0.8 as minimally accurate, 0.8-0.9 as having good accuracy, and > 0.9 as having excellent accuracy, all of which were indicated by Youden’s index.

**Table S8. Test Result Variable(s): the number of phenotypes**

| Positive if Greater Than or Equal To^a^ | Sensitivity | 1 - Specificity |
| --- | --- | --- |
| -1.00 | 1.000 | 1.000 |
| .50 | .986 | .836 |
| 1.50 | .775 | .345 |
| 2.50 | .437 | .091 |
| 3.50 | .218 | .006 |
| 4.50 | .063 | .000 |
| 6.00 | .000 | .000 |

Coordinates of the Curve: The test result variable: “the number of phenotypes” has at least one tie between the positive actual state group and the negative actual state group. *The smallest cutoff value is the minimum observed test value minus 1, and the largest cutoff value is the maximum observed test value plus 1. All the other cutoff values are the averages of two consecutive ordered observed test values.
